# Biomimetic Multi-Responsive Superwettable Materials for Oil–Water Separation

**DOI:** 10.1007/s40820-026-02222-8

**Published:** 2026-05-21

**Authors:** Chengkang Rao, Yan Xin, Zhiguang Guo, Weimin Liu

**Affiliations:** 1 Ministry of Education Key Laboratory for the Green Preparation and Application of Functional Materials, Hubei University, Wuhan, 430062 People’s Republic of China; 2 State Key Laboratory of Silicate Materials for Architectures and School of Materials Science and Engineering, Wuhan University of Technology, Wuhan, 430070 People’s Republic of China

**Keywords:** Smart response materials, Wettability, Oil–water separation, Preparation

## Abstract

Industrial oily wastewater discharges and marine oil spills pose a serious threat to ecosystems. A new strategy for efficient and controllable oil–water separation is provided by smart-responsive wettability materials, owing to their ability to dynamically switch surface wettability in response to external stimuli. Under this premise, we reviewed the research progress and applications of such materials. First, we described the theoretical basis of wettability and the mechanism of oil–water separation. Subsequently, we comparatively analyzed the structural characteristics and separation properties of four types of special wettable materials. We focused on eight categories of smart response wetting materials: temperature, pH, light, electricity, gas, ion, solvent, and multi-response. For each type, we analyzed the response mechanisms, advantages, and limitations in oil–water separation. In addition, we compared the advantages and disadvantages of key preparation techniques such as layer-by-layer self-assembly, electrostatic spinning, and surface-initiated atom transfer radical polymerization. Finally, we summarized the current research status and challenges in the field of smart-responsive wetting materials and looked forward to future development directions.

## Highlights

- By integrating Young's equation, the Wenzel model, and the Cassie–Baxter model, the critical influence of biomimetic micro-/nano-structures and surface chemical regulation on achieving superwettability and smart switching is revealed, providing a theoretical foundation for the design of high-performance separation materials.
- In analyzing stimulus-responsive catalytic cleaning membranes, the four-level synergistic coupling mechanism between the physical barrier effect of the surface hydration layer and the degradative action of catalytically generated reactive oxygen species is elucidated, offering theoretical support for achieving long-term antifouling performance and integrated separation degradation functionality.
- The response principles and oil-water separation performance of temperature, pH, photo, electric, gas, ion, solvent, and multi-responsive materials are systematically reviewed and comparatively analyzed. Notably, a comprehensive evaluation framework is established through comparative tables across multiple dimensions, such as response speed, regulation precision, reversibility, and energy consumption, thereby providing an intuitive reference for material selection.

## Graphical abstract

**Figure:**
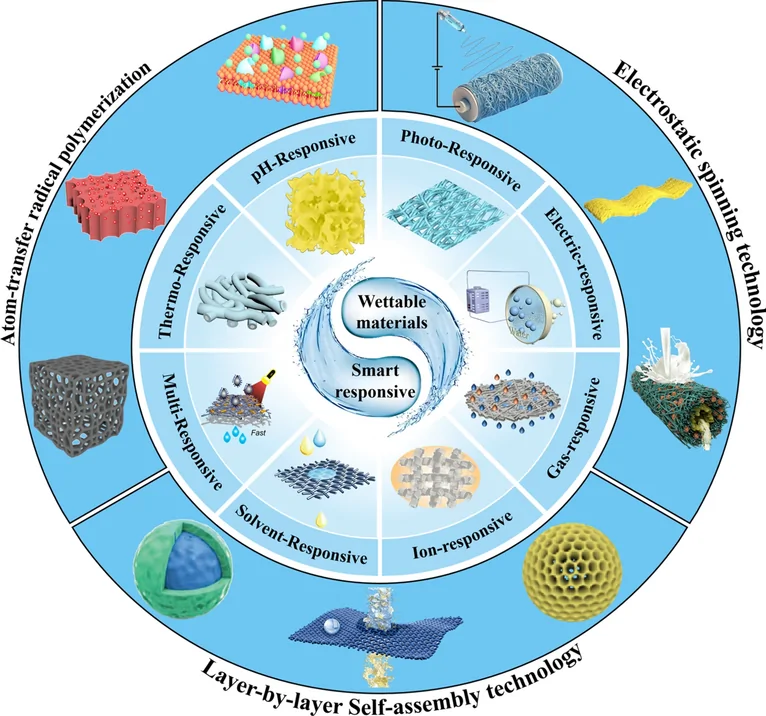

## Introduction

Oil spills [1], industrial oily wastewater [2], and domestic sewage discharges [3] globally introduce large quantities of toxic compounds into water bodies, severely damaging aquatic ecosystems and threatening human health through the water cycle and food chain [4]. This leads to the death of aquatic organisms and deterioration of water quality, especially in the case of oil spills [5], which are difficult to clean up and pose a long-term threat to biodiversity and coastal communities [6]. Conventional water treatment technologies are often expensive, inefficient and may cause secondary pollution and are unable to effectively deal with large-scale pollution problems. Therefore, the development of efficient, environmentally friendly and low-cost water treatment technologies has become an urgent task for the global industrial and scientific community and will ensure the sustainable use of water resources in the future [7]. Traditional water treatment methods, such as in situ combustion, chemical dispersants, mechanical separation, electrolysis, adsorption, centrifugal separation, and biological treatment [8–12], although widely used, suffer from the problems of secondary pollution, low efficiency, high cost, and complicated operation, which limit their large-scale application. Buist et al. first conducted an in situ burning experiment for an oil spill at sea, in which approximately 90% of the spilled oil was consumed after combustion [13]. They then applied a silicone-based chemical herder under icy conditions to facilitate burning, demonstrating that it could effectively increase the thickness of the oil slick and thus be used for emergency response to oil spills in extremely cold marine environments. However, these methods are still insufficient to completely remove oil contamination. More importantly, burning generates large amounts of carbon dioxide, leading to secondary environmental pollution. Therefore, there is an urgent need to develop eco-friendly, efficient, and low-cost oil–water separation technologies [14]. As is well known, significant progress has been made in wettability research, particularly in superhydrophobic/superoleophilic and superhydrophilic/superoleophobic materials, which have demonstrated great potential for oil–water separation. These materials have excellent separation properties, reusability, and low cost, making them ideal for treating oil spills and wastewater. For example, Zhang et al. coated fly ash cenospheres with fluorocarbon resin, micron-sized calcium carbonate, and nanoscale silica and prepared a particulate material with superhydrophobic and superoleophilic properties through a one-step process involving mixing, stirring, and low-temperature drying [15]. This material can rapidly separate oil and water from both static and dynamic oil–water mixtures as well as water-in-oil emulsions, achieving a separation efficiency of up to 99.2% and a flux of 1980 L m^−2^ h^−1^. In addition, it exhibits excellent oil adsorption capacity and strong resistance to acids, alkalis, solvents, high temperatures, and mechanical abrasion, while remaining reusable for at least 40 cycles. Compared with traditional water treatment methods, it offers notable advantages in terms of efficiency, environmental friendliness, and reusability. However, Wei et al. designed a conductive polymer membrane with superhydrophilic and underwater superoleophobic properties, which achieved an oil–water separation efficiency as high as 99.93% [16]. Even after 70 reuse cycles, the separation efficiency remained at 97.18%, demonstrating excellent reusability and stability. With technological advances, wettability modulation technology is expected to further advance the field of oil–water separation [17].

Wettability is a key property of solid surfaces, usually determined by surface chemistry and morphology, and is measured by water contact angle (WCA). Based on the water contact angle, wettability can be categorized as hydrophilic, hydrophobic, superhydrophilic, and superhydrophobic [18]. Superhydrophobicity means that the water droplets hardly touch the surface and is inspired by the “lotus leaf effect”. Organisms such as lotus leaves [19] and water striders [20] demonstrate superhydrophobic behavior in nature, which has inspired researchers to develop artificial superhydrophobic surfaces through micro- and nanostructures or surface chemical modifications. These surfaces possess self-cleaning, water-repellent, and dirt-resistant capabilities. Superhydrophobic surfaces have become a research hotspot in recent years due to their wide range of promising applications, such as self-cleaning materials [21], anti-icing coatings [22], and oil–water separation.

With further research, the flexibility and adaptability of conventional static superhydrophobic surfaces in complex environments show some limitations in responding to changes in the external environment. In contrast, over millions of years of evolution, nature has endowed biological surfaces with highly adaptive features. For example, the ability of starfish [23], chameleons [24], and bacteria [25] to respond to stimuli such as light and temperature has inspired the design of smart materials. Based on these natural properties, scientists have developed smart-responsive surfaces, in particular switchable wettable materials, which can adjust their properties in response to external stimuli (e.g., light, temperature, pH, etc.). These materials are suitable for applications such as drug delivery, cell encapsulation, oil–water separation, microfluidics, and sensors [26]. For example, smart surfaces can precisely control drug release, improve oil–water separation efficiency, and regulate fluid flow [27–32], providing innovative solutions for biomedical and chemical engineering. In a word, smart surface materials not only advance the development of materials science, but also provide new solutions to modern technological challenges, with broad prospects for future applications.

**Fig. 1:**
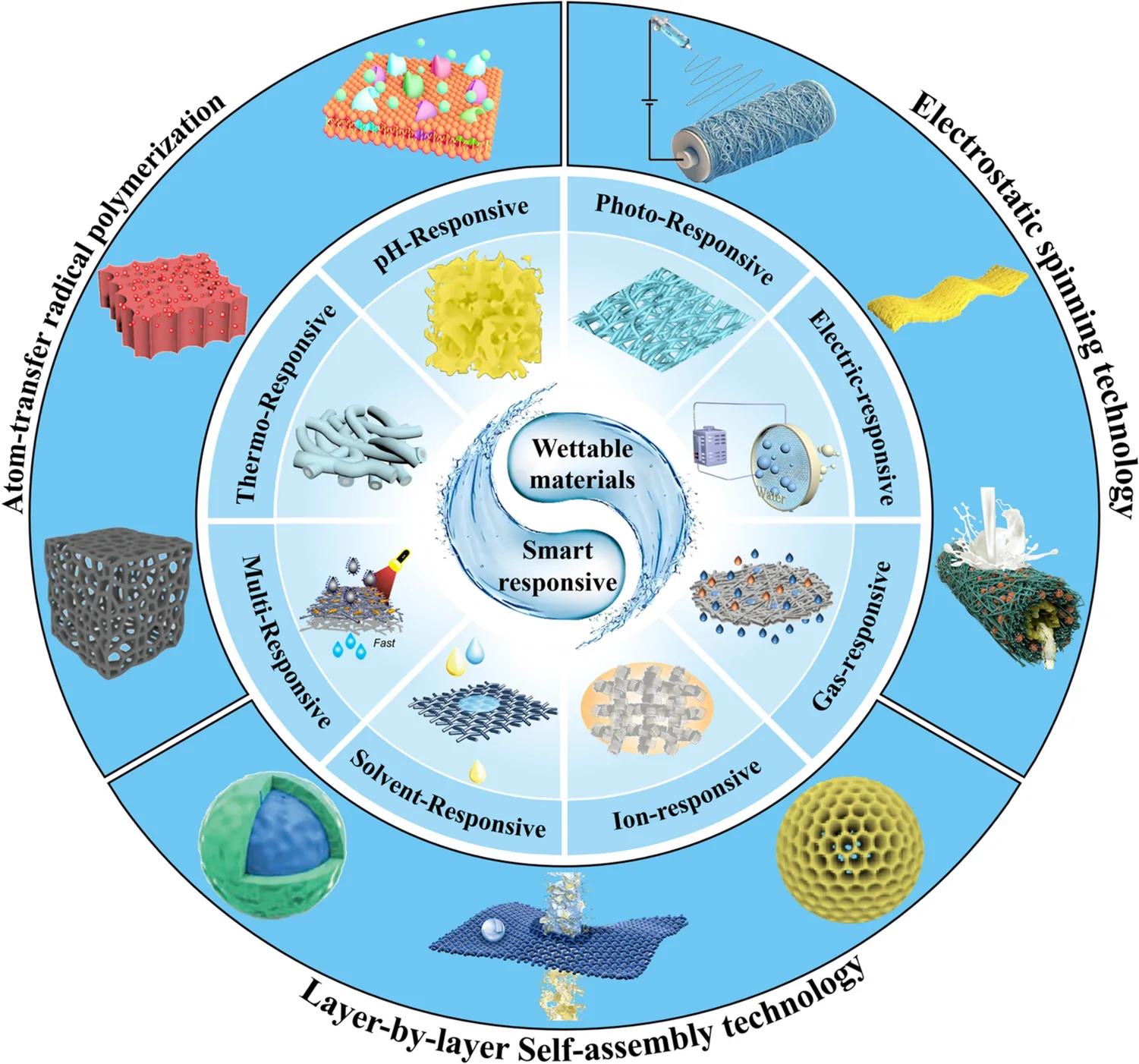
Integrated framework diagram of smart response wettability materials. This figure adopts a concentric circle structure from the outside to the inside, layer by layer, to systematically show the basic logic of the research on smart response wettability materials. The outermost layer is the three major preparation technologies, such as layer-by-layer self-assembly, electrostatic spinning, and surface-initiated atom transfer radical polymerization, which represent the technical means and source driving force for realizing smart wettability. In the middle ring, there are eight types of intelligent responses, including temperature response, pH response, light response, electric response, gas response, ion response, solvent response, and multiple response, which represent the diversified response mechanisms constructed by the preparation methods. The Tai Chi diagram in the center represents the relationship between “smart response” and “wettable materials”, which are mutually promoting and developing together. The principle is that the smart response gives the material the ability to adapt to the environment, and the wettable material provides the basis for the realization of the function. The final fusion is the smart response wettable materials, which can intelligently switch the wettability through different types of stimulus response, thus realizing efficient oil–water separation

Based on the above, we systematically reviewed the research progress of smart-responsive wetting materials. We designed Fig. 1, as the core conceptual framework of this paper, to integrate the research content through a three-layer progressive structure of “preparation mechanism theory”. The Taiji diagram in the inner layer symbolized the mutual promotion of the two core concepts of “smart response” and “wettability”, serving as the theoretical cornerstone of the whole paper and corresponding to Chapter 2, where we also discussed and analyzed the structural characteristics and separation properties of four types of special wettable materials. Around this core, the middle layer specified the intelligent response mechanisms into eight types, such as temperature, pH, and light, corresponding to Chapter 3, in which we comparatively analyzed their response mechanisms, advantages, and limitations in oil–water separation. The outermost layer provided the technological paths to achieve these functions—namely three preparation methods: layer-by-layer self-assembly, electrospinning, and surface-initiated atom transfer radical polymerization—corresponding to Chapter 4 of the thesis. Finally, we summarized the current status and challenges in this material field and looked forward to future research directions.

## Theoretical Basis of Wettability

### Contact Angle

Surface wettability is the ability of a liquid to spread or coalesce on a solid surface, which is determined by a combination of surface morphology and chemical composition [33]. Four states can be classified according to the water contact angle (CA): hydrophilic surfaces (10° < contact angle < 90°), where droplets spread easily; hydrophobic surfaces (90° < contact angle < 150°), where droplets are spherical and do not spread easily; superhydrophilic surfaces (contact angle < 10°), where droplets spread almost completely; and superhydrophobic surfaces (contact angle > 150°), where droplets are nearly spherical and do not spread at all [34]. Similarly, in the case of oil, the same criteria can be used to define oleophilic/oleophobic surfaces as well as superoleophilic/superoleophobic surfaces. It is important to note that while Young’s equation uses 90° as the mathematical demarcation between hydrophilic/hydrophobic, 65° has been proposed as a more realistic threshold for the hydrophilic–hydrophobic transition, taking into account water molecule interactions [35, 36]. For example, Feng et al. prepared a superhydrophobic surface consisting of nanofibers with a water contact angle as high as 171.2° using an amphiphilic polyvinyl alcohol with an intrinsic contact angle of only 72.1° [37]. However, to better illustrate the issue of superwetting, the wetting properties discussed in this paper follow the conventional view held by most, which defines superhydrophobicity as a contact angle greater than 150° and superhydrophilicity as a contact angle of less than 10°. It is this extreme wetting selectivity that has led to the widespread interest in superhydrophilic and superhydrophobic materials in the fields of oil–water separation, environmental protection, wastewater treatment, and oil cleanup.

#### Young’s Equation

**Fig. 2:**
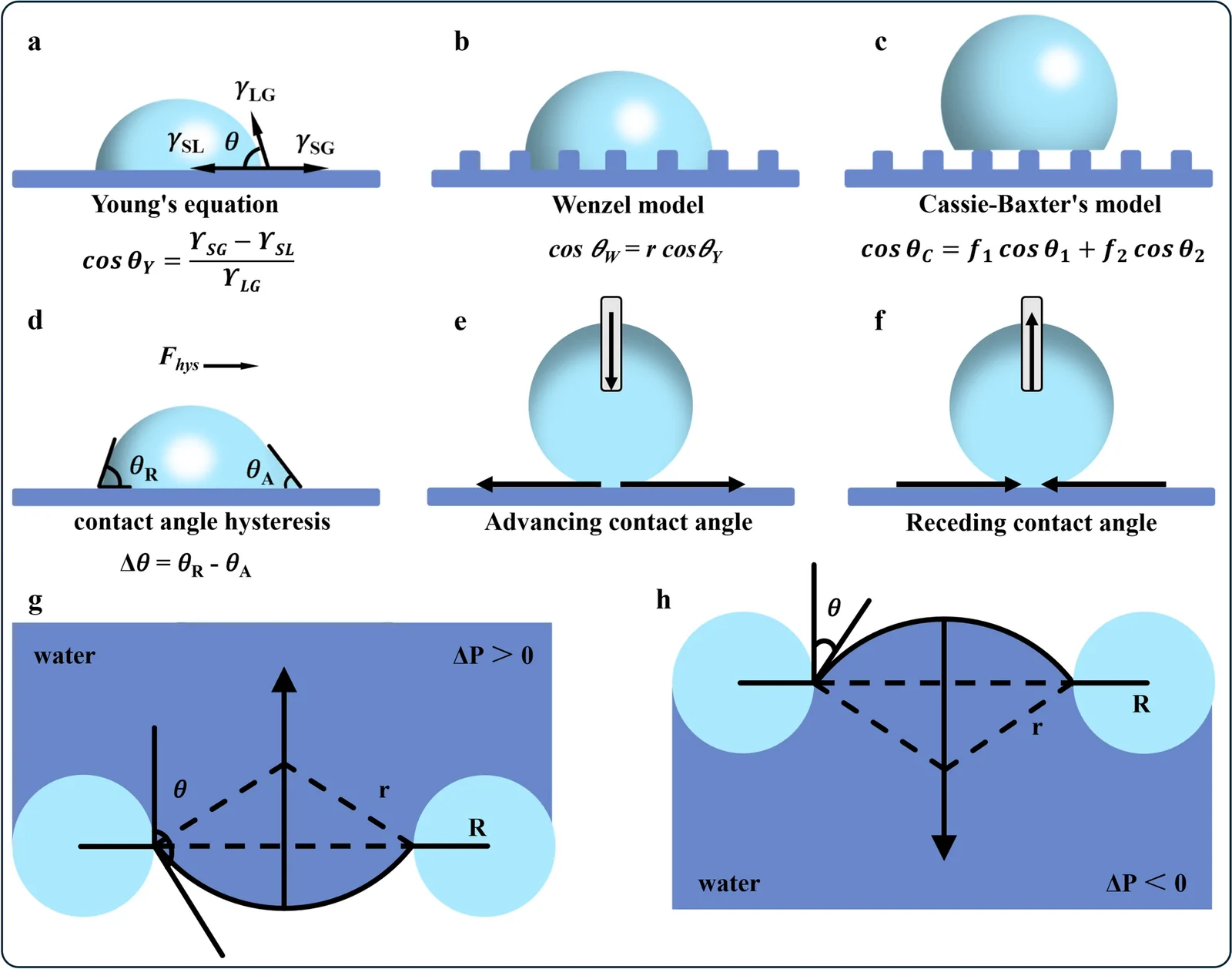
Basic theories controlling wettability: **a** schematic diagram of Young’s equation, **b** schematic diagram of Wenzel’s equation, **c** schematic diagram of Casey’s equation, **d** contact angle hysteresis. Schematic representation of the **e** advancing contact angle and **f** receding contact angle of a horizontal rough surface. **g** When the contact angle of the liquid in air is greater than 90°, the liquid cannot penetrate the porous surface. **h** When the contact angle of the liquid in air is less than 90°, the liquid can spontaneously penetrate the porous surface

In 1805, Thomas Young introduced an equation (Fig. 2a) describing the equilibrium between surface tension and contact angle on a perfectly uniform and smooth horizontal surface [38]:

1 $$\Upsilon_{{{\mathrm{LG}}}} \cos \Upsilon_{Y} = \Upsilon_{{{\mathrm{SG}}}} - \Upsilon_{{{\mathrm{SL}}}}$$

where $\Upsilon_{{{\mathrm{SL}}}}$, $\Upsilon_{{{\mathrm{SG}}}}$, $\Upsilon_{{{\mathrm{LG}}}}$ are the solid–liquid, solid–gas, and liquid–gas interfacial tensions, respectively, and *θ*_*Y*_ is the angle between the tangent line of the droplet surface and the horizontal plane. Bounded by 90°, contact angle < 90° is the hydrophilic state and contact angle > 90° is the hydrophobic state [17]. Young’s equation suggests that the contact angle depends only on the three-phase interfacial tension. However, there are microscopic inhomogeneities on the actual surface, and factors such as droplet volume and pH affect the contact angle measurements.

#### Wenzel Model

Since perfectly uniform and smooth surfaces do not exist in reality, Wenzel introduced the concept of roughness (*r*) based on Young’s equation to better represent real surfaces.

2 $$r = \frac{{A_{{{\mathrm{actual}}}} }}{{A_{{{\mathrm{projection}}}} }}$$

where *A*_actual_ is the total actual area of the material surface and *A*_projection_ is the projected area. Wenzel’s equation (Fig. 2b) is given to calculate the contact angle on a rough surface [39].

3 $$\cos \theta_{w} = \frac{{r\left( {{\Upsilon}_{{{\mathrm{SG}}}} - {\Upsilon}_{{{\mathrm{SL}}}} } \right)}}{{{\Upsilon}_{{{\mathrm{LG}}}} }}$$

where *r* is defined as the roughness, which is the ratio of the actual solid–liquid interfacial contact area to the apparent contact area (*r* ≥ 1), and *θ*_*W*_ is the contact angle of the rough surface in the Wenzel state. The relationship between the contact angle in the Wenzel model and the contact angle in the Young model can be expressed by the following equation [40]:

4 $$\cos \theta_{W} = r \, \cos \theta_{Y}$$

where *θ*_*W*_ is the apparent contact angle of the rough surface in the Wenzel state. Since all real surfaces have a rough structure (*r* > 1), roughness amplifies the intrinsic wettability of the material: hydrophilic surfaces are more hydrophilic, and hydrophobic surfaces are more hydrophobic. It should be noted that the Wenzel model is not applicable to porous or chemically complex surfaces.

#### Cassie–Baxter’s Model

The Wenzel equation has difficulty in explaining certain superhydrophobic phenomena in nature, such as the very low adhesion of the surface of the lotus leaf. If a liquid completely infiltrates a rough surface, only a solid–liquid interface exists, and increased roughness increases the contact area, which theoretically should lead to a rise in adhesion, which contradicts the actual low adhesion of the lotus leaf. To explain this phenomenon, Cassie and Baxter proposed the concept of air-gas pockets: the liquid does not fully infiltrate the rough structure, but traps the gas in the grooves, forming a composite solid–liquid and liquid–gas interface [41]. Based on this, they proposed the Cassie–Baxter equation (Fig. 2c).

5 $$\cos \theta_{C} = f_{1} \cos \theta_{1} + f_{2} \cos \theta_{2}$$

where ${f}_{1}$ denotes the contact area fraction of a single component in a composite interface, with different components distinguished by subscripts. *θ*_1_ is the intrinsic contact angle of ${f}_{1}$. Similarly, *θ*_2_ is the intrinsic contact angle of ${f}_{2}$. For composite interfaces of water and solid–gas, one group is considered as a gas–liquid interface. Let ${f}_{2}$ be the area fraction of the liquid–gas interface and ${f}_{1}$ be the area fraction of the liquid–solid interface. The sum of ${f}_{1}$ and ${f}_{2}$ is equal to 1. So ${f}_{2}$ can also be expressed as:

6 $$f_{2} = 1 - f_{1}$$

This equation simplifies the calculation of wetting states on surfaces such as lotus leaves (commonly referred to as the Cassie state, or C state). In other words, when the water touches the surface, it does not completely wet the material and air is left between it and the surface [42]. The wetting interface is a water–solid–air composite wetting interface.

#### Contact Angle Hysteresis

Young’s equation, Wenzel’s model, and Cassie–Baxter’s model describe the static contact angle on chemically homogeneous smooth surfaces, fully infiltrated rough surfaces and composite contact rough surfaces, respectively [43]. However, real surfaces always have inhomogeneities in chemical composition or defects in physical structure, which inevitably lead to fluctuations in the static contact angle [44]. It is these “deviations” from the ideal that lead to contact angle hysteresis. For a droplet with a rough surface and chemical inhomogeneity, imagine that you continue to add water to a droplet on a surface, the volume of the droplet increases, but the contact area at the bottom remains temporarily unchanged until a critical point, when the droplet begins to move forward, and at this point the contact angle is the advancing contact angle (*θ*_A_) (Fig. 2e). However, if you draw water from a surface droplet, the droplet volume decreases, but the contact area at the bottom stays the same for a while until a critical point where the droplet starts to move backward, the contact angle at this point is the receding contact angle (*θ*_R_) (Fig. 2f) [45]. The difference between them is the contact angle hysteresis, *Δθ* = *θ*_A_—*θ*_R_. The hysteresis resistance is the adhesion force caused by the contact angle hysteresis in the opposite direction of the droplet motion (Fig. 2d).

7 $$F_{{{\mathrm{hys}}}} = 2\gamma R_{0} \left( {\cos \theta_{{{\mathrm{Ro}}}} - \cos \theta_{{{\mathrm{Ao}}}} } \right)$$

where *θ*_A0_ and *θ*_R0_ are the position-dependent advancing and receding contact angles, respectively, *R*_0_ is the characteristic radius of the droplet, and *Υ* is the surface tension of water.

### Mechanism of Oil–Water Separation

The core principle of oil–water separation is to utilize the different physicochemical properties of the oil phase and the water phase to realize the separation of oil–water mixtures. In the process of oil–water separation, the interfacial effect between the oil and water phases is very significant [46]. Therefore, when designing materials suitable for oil–water separation, we need to focus on the surface structure and intrusion pressure, which are two physical parameters that affect the separation performance. The surface structure is mainly reflected in the pore size and porosity of the material. High porosity provides more transportation channels for fluids and is the physical basis for obtaining high permeation flux. Under the premise of ensuring that the dispersed phase (e.g., oil or water droplets) can be effectively intercepted, appropriately increasing the pore size can significantly reduce the resistance of the fluid when it passes through, thus obtaining a high separation flux [47]. However, too large a pore size can complicate the oil–water separation process by preventing the formation of stable water-pore structures that are more susceptible to mechanical or hydrodynamic damage [48]. Ideal separation materials usually need to strike a balance between high porosity (to ensure high flux) and suitable pore size (to ensure selectivity and interfacial stability): using high porosity to enhance permeation velocity, while controlling the pore size to intercept the dispersed phases and maintain a stable interfacial structure, ultimately realizing efficient and stable oil–water separation.

The intrusion pressure difference (*ΔP*_*C*_) is the maximum pressure exerted by the fluid on the surface before penetrating the pore space, i.e. the pressure exerted by the droplet on the substrate [49]. Thus the smaller the droplet, the higher the pressure. Its formula can be expressed by the Young–Laplace equation [50]:

8 $$\Delta P_{c} = - \frac{{2\Upsilon_{L} \cos \theta }}{R}$$

where *Υ*_*L*_ is the surface tension of the liquid, *θ* is the angle of contact from the inside of the liquid, is the angle formed by the liquid–gas interface with the solid surface on a flat substrate, and *R* denotes the pore radius. From the previous Laplace equation, the interfacial wettability of the material has a significant effect on the osmotic pressure [51]. Precisely, when *θ* > 90°, the osmotic pressure value is positive (*ΔP*_*C*_ > 0), the material surface can withstand a certain amount of external pressure, in the stationary or low-pressure state, the droplets will tend to maintain a spherical shape, stay on the surface of the material (i.e., the “rolling” state), and need to apply an external force in order to make the droplets penetrate the surface of the material. However, when *θ* < 90°, the osmotic pressure value is negative (*ΔP*_*C*_ < 0), the surface cannot maintain a composite interface, and the liquid does not need external force and can even spontaneously penetrate the material surface, resulting in rapid surface wetting and spreading [52].

Therefore, the surface of a material with superhydrophobic/superoleophilic properties is actually free of oil. Because the material is lipophilic, the contact angle of oil on the surface is small and the osmotic pressure facing the oil phase is negative. The oil will quickly spread out and penetrate into the pore channels to achieve rapid separation. Meanwhile, water is blocked from the surface [53]. However, it is different for the surface of materials with underwater superoleophobic properties. Usually the underwater superoleophobic surface is hydrophilic, and the air will be replaced by water to form a stable water film (hydration layer) on the surface. When an oil droplet contacts this water film, the oil droplet is actually in contact with a layer of water molecules on the surface of the solid, rather than in direct contact with the solid [54]. In this solid–liquid–liquid system, the oil droplet presents a large contact angle, so that with *θ* > 90° and *ΔP*_*C*_ > 0, the oil droplet cannot penetrate.

However, the separation mechanisms described above are mainly for floating or dispersed oil (droplet size > 100 μm). In practice, oil–water mixtures often exist in more complex emulsion forms, which are significantly more difficult to separate. An emulsion is a stable system formed by the dispersion of one liquid in the form of tiny droplets in another immiscible liquid. The core difference between emulsions and normal oil–water mixtures is twofold: firstly, the droplet size is extremely small (typically < 20 μm), which makes it difficult to naturally stratify on standstill [55]. Secondly, the stability is extremely high. Due to the presence of surfactants and other emulsifiers, a protective film is formed at the droplet interface, preventing the droplets from merging. According to the different dispersing phases, emulsions are mainly divided into two categories: water-in-oil (W/O) and oil-in-water (O/W) [56]. W/O emulsions are characterized by their continuous phase being the oil phase, and the water droplets are dispersed in the oil phase to become a discontinuous phase. On the contrary, O/W emulsions show the opposite phase morphology, with water as the continuous phase and oil droplets suspended in it as the dispersed phase. The stabilizing mechanism lies in the adsorption of emulsifier molecules at the oil–water interface: in W/O emulsions, lipophilic substances such as asphaltene form an interfacial film to reduce the interfacial tension; in O/W emulsions, hydrophilic substances such as mineral salts maintain the dispersion of droplets through electrostatic interaction [57]. Separation of emulsions cannot rely on wettability differences alone, but often requires emulsion breaking pretreatment (e.g., chemical emulsion breaking, physical emulsion breaking) to destabilize the emulsion [58–60] and then combined with membrane separation technology to achieve efficient separation.

### Superhydrophobic/Superoleophilic Materials

Superhydrophobic/superoleophilic materials are typical oil-removing materials. The oil phase diffuses extremely easily and is effectively absorbed in the case of porous bulk materials and penetrates smoothly in the case of porous filtration materials. In contrast, the water phase is strongly repelled. In this way the oil can be successfully separated from the oil–water mixture [61]. It is well known that the formation of surface wettability is not determined by a single factor, but by a combination of factors such as surface chemistry and topography [62]. In view of this, there are two well-established strategies that can be employed in the preparation of superhydrophobic/superoleophilic materials. First, the superhydrophobicity and superoleophilicity of the material can be enhanced by carefully constructing rough structures on the otherwise hydrophobic surface to increase the roughness of the surface. Second, the rough surface is modified with chemicals that have low surface energy. This adjusts the surface chemistry of the material so that it is more favorable for adsorption of the oil phase and repulsion of the water phase [63]. So far, many strategies have been developed and applied to design and fabricate “oil-removing” materials.

**Fig. 3:**
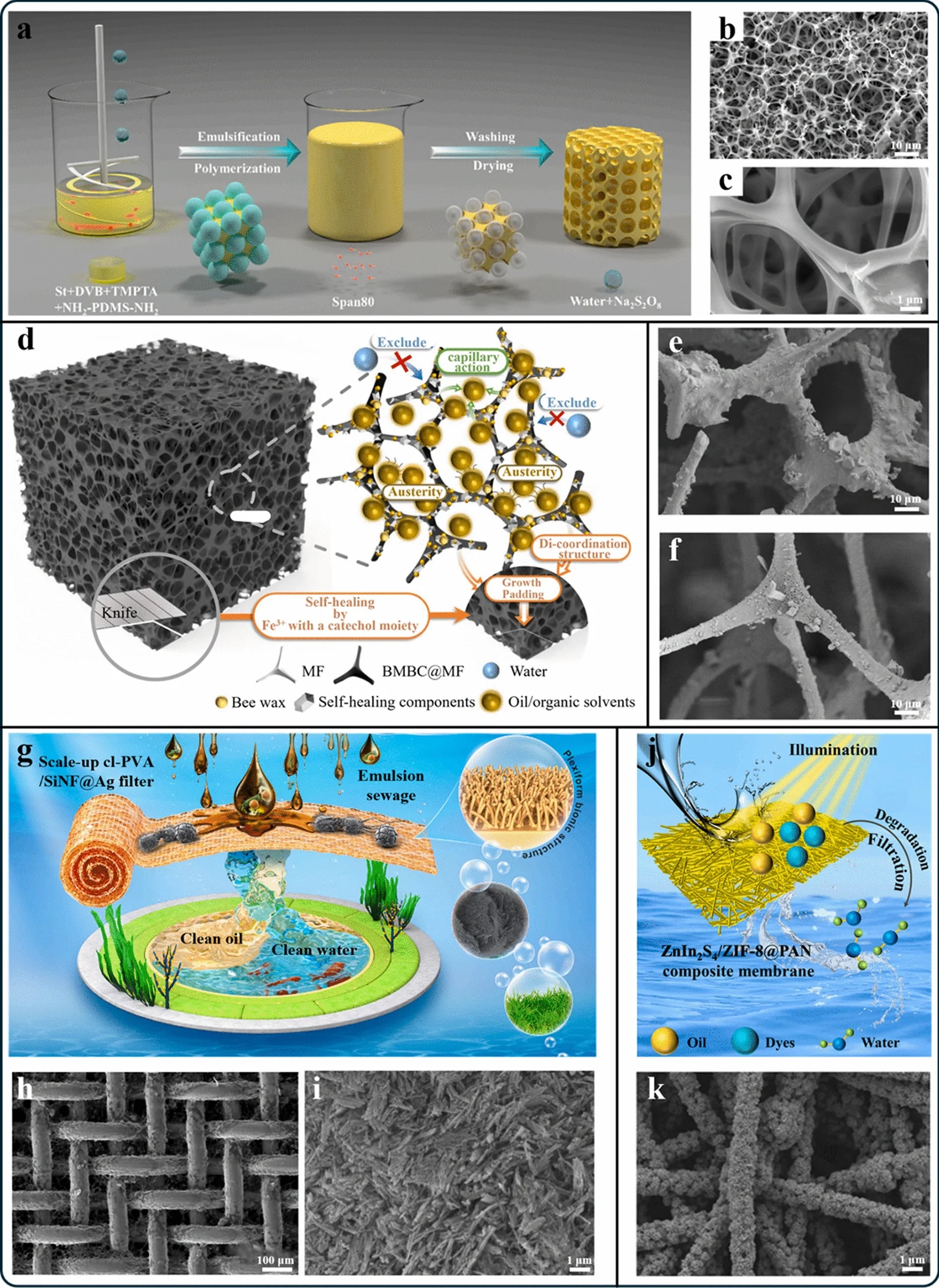
**a** Schematic depicting the fabrication process of superelastic superhydrophobic foams. **b** SEM image of P(St_40_-D_30_-T_10_)/NPN_20_. **c** High-magnification image of P(St_40_-D_30_-T_10_)/NPN20. Reproduced from Ref. [64] with permission, copyright 2024, Elsevier B.V. **d** Schematic of adsorption mechanism and self-healing mechanism of SH-Wax@BMBC@MF on oil/organic solvent and water. SEM images of SH-Wax@BMBC700BMBC suspension with MF concentrations of **e** 3 mg mL^−1^,** f** 5 mg mL^−1^. Reproduced from Ref. [65] with permission, copyright 2024, Elsevier B.V. **g** Schematic diagram of a new filter for separating marine-emulsified wastewater. **h–i** SEM image of cl-PVA/SiNF@Ag–Cu containing 0.6 g of SiNF. Reproduced from Ref. [77] with permission, copyright 2025, Elsevier B.V. **j** Schematic diagram of nanofiber composite membrane for separation of emulsified oily wastewater. **k** SEM image of ZnIn_2_S_4_/ZIF-8@PAN. Reproduced from Ref. [78] with permission, copyright 2025, Elsevier B.V

Super-impregnated materials are in the spotlight for their oil–water-selective separation ability, two-dimensional porous materials are limited in their application, and three-dimensional porous materials are suitable for large-scale separation but suffer from the problems of difficult emulsion separation, complicated preparation, and poor stability. Zhao et al. used high internal phase emulsion polymerization to design and prepare a polystyrene-based porous material (Fig. 3a). This material exhibits superelasticity and superhydrophobicity/superoleophilicity. The authors introduced flexible and hydrophobic aminopropyl-capped polydimethylsiloxane (NH_2_-PDMS-NH_2_) chain segments into the rigid styrene–divinylbenzene copolymer via a 1,4-conjugate addition reaction. This created an open-pore, interconnected porous network with fluid-absorbent channels (Fig. 3b, c). Consequently, the material exhibited simultaneously improved mechanical and hydrophobic properties, with the water contact angle increasing from 141.2° to 152.2°. In addition, the material can continuously and efficiently separate immiscible oil–water mixtures and O/W emulsions in harsh environments, with a maximum separation flux of 5453 L m^−2^ h^−1^ bar^−1^ and a separation efficiency of more than 98.4%, which shows great potential for large-scale treatment of complex oily wastewater [64]. Although the preparation method of the material is relatively simple, the raw materials used, such as NH_2_-PDMS-NH_2_, may be costly, which limits its large-scale application. Hu et al. prepared a superhydrophobic/superoleophilic biochar-based foam (Wax@BMBC@MF) consisting of ball milled biochar (BMBC) and natural beeswax-coated melamine foam (MF) by a bio-inspired functionalization method and further endowed with self-healing functionality (SH-Wax@BMBC@MF) (Fig. 3d), which is able to efficiently adsorb and separate the oil–water mixtures (Fig. 3e, f) [65]. The material is easy to prepare, low cost, and suitable for large-scale application, providing a promising solution for offshore oil spill treatment. However, although this foam shows advantages in oil–water separation efficiency and cost-effectiveness, its mechanical strength and separation precision for microemulsions still leave room for improvement. To further enhance performance, Wang et al. developed an electrospun nanofiber/covalent organic framework (ETCNF/COF) composite superwetting membrane. A fibrous network was first constructed by electrospinning, followed by the in situ-controlled growth of vertically aligned COF nanorods on the fiber surface. Subsequently, surface functionalization was achieved via thiol-ene click chemistry, yielding two types of membranes. The ETCNF/COF-ODM membrane, modified with octadecanethiol, exhibited superhydrophobic/superoleophilic properties and could efficiently separate water-in-oil emulsions. In contrast, the ETCNF/COF-CHA membrane, modified with cysteamine hydrochloride, displayed superhydrophilic and underwater superoleophobic properties, enabling efficient separation of oil-in-water emulsions. The hierarchical porous network, integrating rapid transport through macropores with selective passage through mesopores, improved both water flux and emulsion separation efficiency, while also providing high mechanical strength, solvent resistance, and stable performance over multiple reuse cycles. Experimental results showed that both membranes achieved separation efficiencies above 99% for various emulsions and also exhibited low adhesion and self-cleaning properties.

Ideal oil-removing materials are usually considered to have superhydrophobic and superoleophilic properties, high oil absorption capacity and low water absorption, low density, environmentally friendly, self-propelled discharge of external energy, and good recoverability of various oils/organics. A variety of popular oil-removing materials are available, including fabric-based materials that are soft, bendable, and suitable for a wide range of complex surfaces and shapes [66]; sponge-based materials with good adsorption properties and elasticity for stable oil-removing under varying pressures [67]; metal mesh-based materials with high strength and durability for large-scale industrial applications [68]; and carbon and its derivatives, such as activated carbon and graphene [69], which have a high specific surface area and excellent adsorption performance [70, 71]. In addition, particulate oil-removing materials also play an important role in some specific application scenarios. Although this type of “oil-removing” material exhibits high flux and efficiency when separating the oil phase, its inherent oleophilicity makes its surface prone to oil contamination, leading to a decline in separation performance over time. Additionally, because it can only separate the oil phase, its efficiency is limited when dealing with oil–water mixtures that have a high water content. These limitations have driven researchers to explore materials that can actively resist oil fouling or switch separation modes on demand.

### Superoleophobic/Superhydrophilic Materials

Unlike traditional oil-removing materials, superhydrophilic/superoleophobic “water-removing” materials can selectively separate water from oil–water mixtures, which shows significant advantages: (1) Superoleophobicity effectively prevents oil from adhering, overcomes the problem of oil pollution, extends the service life of the material, and at the same time improves the efficiency of oil recovery and the recyclability of the material, enhancing the economy and sustainability of its application [72, 73]. (2) The high-efficiency separation driven by gravity is particularly outstanding. Since the density of oil is usually less than that of water, water can quickly pass through the “water-removing” material, while oil is effectively blocked. This makes the material extremely efficient when dealing with oily water mixtures with high water content and is widely used in environmental protection, industry, and oil recovery [74, 75]. (3) The unique advantages in oil spill treatment should not be ignored. Superhydrophobic materials can quickly expel water and concentrate oil in marine oil spill accidents, greatly improving the oil recovery rate and reducing environmental pollution [76]. Based on the above advantages, more and more “water-removing” materials have been designed and manufactured. Inspired by the multilayered structure of grasses, Wang et al. constructed a novel filter with a clumped–structure hydrogel interface. They used seafoam-derived silica nanofibers (SiNF) as the support and cross-linked polyvinyl alcohol (cl-PVA) hydrogel as the coating (Fig. 3g). The incorporation of SiNF can build up a homogeneous nanofiber roughness structure, and combined with the hydrogen-bonded crosslinking of hydrogel coating, it lays a key structural foundation for the superhydrophilic/underwater superoleophobic properties and high stability and efficient oil–water separation performance of the material. The addition of SiNF can build up a uniform nanofiber rough structure combined with the hydrogen-bonded crosslinked hydrogel coating, which lays a key structural foundation for the superhydrophilic/underwater superoleophobic properties, high stability, and high efficiency of oil–water separation (Fig. 3h, i). By assembling the nanofibers on the self-designed equipment, the emulsified wastewater can be purified continuously (treatment capacity up to 576.00 L/day), and the separation efficiency of the complex oil-containing emulsion under gravity alone is up to 99.7%, and the oil can be recovered at the same time [77]. However, this filter relies on a stable hydration layer, which may be challenged in extremely dry environments, and its mechanical durability is limited (loses separation ability after 20 sandpaper rubs). Zhan et al. constructed a bifunctional seaweed-like ZnIn_2_S_4_/ZIF-8@PAN nanofiber composite membrane, which was prepared by a combination of electrospinning and in situ synthesis techniques (Fig. 3j). The rough structure, abundant hydroxyl groups and capillary force provided by the alginate ZnIn_2_S_4_ make the composite membrane superhydrophilic (water contact angle of about 0°), superoleophobic (oil contact angle of up to 155.9° underwater), and ultra-low oil adhesion (Fig. 3k) [78]. The composite membrane has good stability and reusability in harsh environments and has great potential in the treatment of complex emulsified oily wastewater.

Nonetheless, there are limitations to “water-removing” materials, especially in the treatment of thick oil–water mixtures, where the high density of the thick oil may settle and adhere to the membrane surface, forming a barrier layer that interferes with the permeation of the water and reduces the efficiency of the separation [79]. In addition, it is difficult to prepare superhydrophilic/superoleophobic materials because they require both strong hydrophilicity and strong oleophobicity in chemistry, which results in high development costs and complex processes, limiting their wide application in certain fields. Therefore, there is an urgent need for advanced materials with convertible wettability between “oil-removing” and “water-removing” to realize on-demand and efficient separation of oil–water mixtures and even emulsions.

### Janus Materials

In practical applications, single wettability materials often exhibit large limitations due to the diversity of environmental conditions. To address this problem, it is particularly important to develop materials with switchable wettability. In recent years, Janus materials have garnered significant attention owing to their asymmetric wettability and switchable properties during oil–water separation. These materials possess the two-sided property of being hydrophilic on one side and oleophobic on the other, which makes them act as a tunable barrier in the oil–water separation process [80–83].

In addition to oil–water separation, Janus materials also possess the unique ability for unidirectional liquid transport, making them suitable for applications such as smart liquid manipulation, interfacial regulation, and sensing. There are two main strategies for the preparation of Janus materials [87]. The first is asymmetric combination, where two materials with different surface properties are directly integrated to form a dual-faced structure, such as combining a hydrophobic copper mesh with a superhydrophobic cotton layer to create a Janus system [88, 89]. However, this method is limited by the inherent properties of the raw materials and may have limited effectiveness in applications such as oil–water separation. The second strategy is asymmetric modification of a substrate, where different functional groups are selectively introduced onto a single substrate to impart different wettabilities to its two sides [90]. Building on this, researchers have developed strategies for constructing layered porous Janus materials, enabling precise control over the microstructure. For example, electrospinning allows for the sequential deposition of fiber layers with different diameters and thicknesses, thereby adjusting the pore size and optimizing separation efficiency and material stability [91, 92]. Similarly, by depositing hydrophilic carbon nanotubes onto the surface of a hydrophobic polyvinylidene fluoride (PVDF) membrane, layers with different wettability, thermal conductivity, or electrical conductivity can be constructed [93]. This not only optimizes the material’s wetting properties but also enhances its mechanical strength and electrical conductivity. Although these methods offer advantages in terms of scalability and cost, achieving the optimal combination of layers remains challenging.

**Fig. 4:**
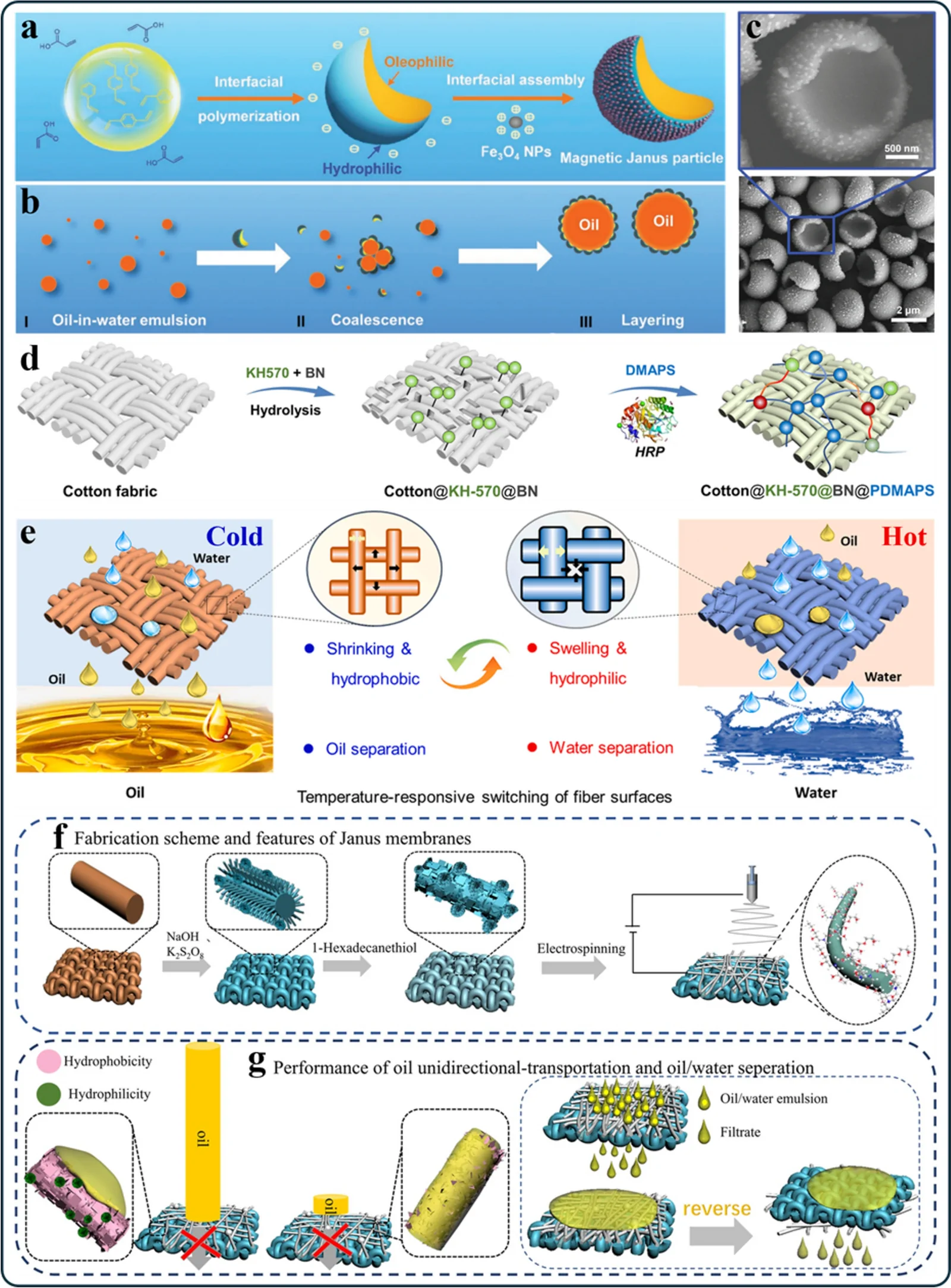
**a** Schematic diagram of the preparation of claw-shaped magnetic Janus particles. By designing the copolymerization of hydrophilic/lipophilic monomers at the oil–water interface of liquid droplets, claw-shaped poly(styrene-divinyl benzene) (PSDVB), poly(acrylic acid) (PAA) Janus particles with convex hydrophilic/concave lipophilic properties were formed. Positively charged Fe_3_O_4_ nanoparticles were modified on the convex surface of Janus particles to form magnetic Janus particles. **b** Schematic diagram of micro-oil droplets separated by Janus particles. These Janus particles first coalesce the tiny oil droplets and then act as surfactants to stabilize the larger oil droplets. **c** SEM images of PSDVB ⊃ PAA magnetic Janus particles. Reproduced from Ref. [84] with permission, copyright 2018, Wiley. Schematic diagram of constructing temperature-responsive composite textiles of **d** KH-570 @BN@PDMAPS and **e** its applications for oil–water separation. Reproduced from Ref. [85] with permission, copyright 2023, Elsevier BV. **f** Preparation scheme and characterization of Janus membranes. **g** Unidirectional transportation of oil and oil–water separation properties. Reproduced from Ref. [86] with permission, copyright 2022, American Chemical Society

While conventional methods (e.g., skimming, centrifugation, gravity separation) and emerging superwettable materials (e.g., membranes, sponges) are effective in handling floating and dispersed oils, and even surfactant-stabilized emulsions, they have limited effectiveness in separating micron-sized droplets of fine oils, which are not stabilized by surfactants and are smaller than 20 microns in size. Wang et al. prepared magnetic Janus particles with convex hydrophilic/concave lipophilic surfaces (Fig. 4a) by emulsion interfacial polymerization and selective surface assembly, which successfully achieved efficient separation of microscale oil droplets from water. The process was carried out by emulsion interfacial polymerization between hydrophilic acrylic acid (AA) and lipophilic styrene/divinylbenzene (St/DVB) monomers, followed by electrostatic assembly of Fe_3_O_4_ nanoparticles onto the convex surface of the Janus particles. Fe_3_O_4_ nanoparticles exist in an aggregated form on the convex surface of the particles and do not completely cover the entire surface, ensuring the hydrophilic–lipophilic characteristics of the particles and rapid magnetic response in aqueous solution (Fig. 4c), and by capturing oil droplets like surfactants and inducing them to aggregate into larger droplets, the oil droplets are stabilized (Fig. 4b) [84]. Its advantages lie in precise micro- and nanostructure modulation and efficient magnetic response performance, but there are limitations in macroscopic continuous separation and large-area applications. Similarly, the KH-570@BN@PDMAPS composite fabrics prepared by Wang et al. possessed Janus-like bifacial properties, with surface wettability reversibly switching between hydrophilicity and hydrophobicity by temperature regulation. Specifically, by introducing KH-570 and BN to build up the hydrophobic surface and then grafting the temperature-responsive polymer PDMAPS by enzymatic polymerization (Fig. 4d), the material becomes hydrophobic (oil passes through) when the temperature is lower than 28 °C and hydrophilic (water passes through) when the temperature is higher than 28 °C, which achieves highly efficient oil–water separation with a separation efficiency of 95% (Fig. 4e) [85]. Its advantages are good scalability, cyclic stability, and easy-to-operate separation, but it is more simplified in terms of precise design of microstructure and intelligent manipulation of magnetic response. In contrast, Cheng et al. designed an innovative “oil diode” Janus membrane for efficient and environmentally friendly oil–water separation. The membrane was coated with a thin layer of superhydrophilic polylactic acid (PLA) on a superhydrophobic-modified copper mesh by electrostatic spinning, combining environmental friendliness with unidirectional oil transport properties (Fig. 4f, g). The key innovation of the membrane is the introduction of a partially hydrophilic “bud-like” micro-nanostructure on the hydrophobic side, which combines the synergistic effect of material properties and surface wettability to dramatically increase the Laplace pressure and achieve oil intrusion pressures of up to 12 kPa [86]. The membrane showed excellent performance in treating stabilized W/O emulsions containing surfactants, achieving a permeability of 2993 L m^−2^ h^−1^ bar^−1^ and a separation efficiency of 99.6%. In comparison, this membrane has made significant breakthroughs in the design of microstructure and the enhancement of Laplace pressure, providing new ideas and directions for the development of environmentally friendly oil–water separation technology.

However, the Janus material still has some shortcomings, and the inability to continuously separate oil and water is one of its major drawbacks. During the separation process, the material needs to be mechanically or manually rotated to adjust the separation interface to ensure the effective separation of oil or water, which greatly limits the convenience and efficiency of its practical operation [94–96]. In addition, the complexity of the preparation process and the low separation efficiency are factors that have also hindered the popularization of Janus materials in applications. However, smart-responsive materials represent another pathway in the development from static, single wettability toward dynamically tunable wettability. In contrast, smart-responsive materials achieve adaptive wettability switching on a single surface in response to external stimuli, without the need for any external mechanical intervention, thereby enabling the continuous separation of oil–water mixtures or emulsions. It is worth noting that these two approaches are gradually converging. For example, this can be achieved by introducing responsive polymers into Janus structures to construct “smart Janus membranes” [97] or by leveraging the asymmetric modification strategy of Janus materials to enhance their response sensitivity. This synergistic design provides an important concept for developing a new generation of efficient and controllable oil–water separation materials.

### Bioinspired Wettability Materials

**Fig. 5:**
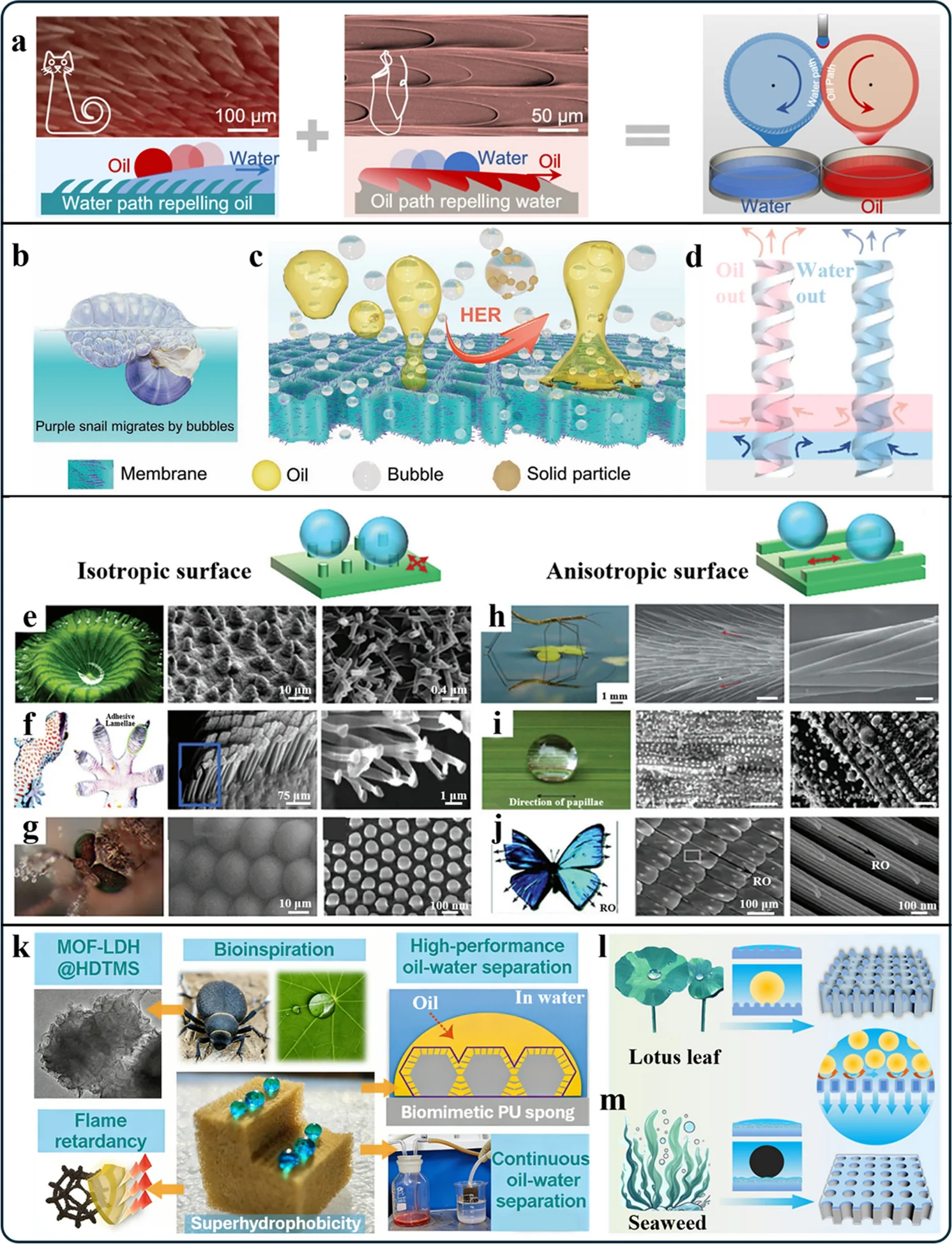
**a** Schematic illustration of the oil–water microdroplet separation process achieved by rotating dual-biomimetic (cat tongue and Nepenthes pitcher plant) superwetting gears. Reproduced from Ref. [119] with permission, copyright 2023, Nature. Biomimetic bubble-mediated dynamic oil removal strategy: **b** the purple snail floats driven by bubbles. **c** Biomimetic bubble-mediated morphological transformation enables dynamic oil removal. Reproduced from Ref. [120] with permission, copyright 2023, Wiley. **d** Schematic illustration of selective separation of individual liquids by placing biomimetic spring microchannels (SMC) prefilled with water or oil onto an oil–water-CCl_4_ mixture. Reproduced with permission. Reproduced from Ref. [121] with permission, copyright 2025, Wiley. Photographs and SEM images of underwater SHS, SLIPS, and smooth surfaces. More specifically, **e** photographs and SEM images of lotus leaves. Reproduced from Ref. [99] with permission, copyright 2009, Royal Society of Chemistry. **f** Gecko setae. Reproduced from Ref. [100] with permission, copyright 2005, Proceedings of the National Academy of Sciences. **g** Compound eye of a mosquito. Reproduced from Ref. [101] with permission, copyright 2007, Wiley. **h** Water tumbling over legs. Reproduced from Ref. [102] with permission, copyright 2010, Cambridge University Press. **i** Rice leaves. Reproduced from Ref. [103] with permission, copyright 2007, Elsevier BV. **j** Butterfly wings. Reproduced from Ref. [104] with permission, copyright 2007, Royal Society of Chemistry. **k** Inspired by desert beetles and lotus leaves, a biomimetic micro-/nano-composite structure was constructed by in situ growth of metal–organic framework-derived layered double hydroxide (MOF-LDH) on the surface of a polyurethane (PU) sponge. After grafting MOF-LDH with hexadecyltrimethoxysilane (HDTMS), FPUF@MOF-LDH@HDTMS can be used for efficient continuous oil–water separation. Reproduced from Ref. [122] with permission, copyright 2023, Elsevier BV. **l** Lotus leaf-inspired design of superwetting membranes achieved by constructing nanostructures on hydrophilic membrane surfaces. Reproduced from Ref. [114] with permission, copyright 2011, AIP Publishing. **m** Seaweed-inspired design of superwetting membranes achieved by forming a stable hydration layer on the membrane surface. Reproduced from Ref. [118] with permission, copyright 2015, Wiley

The surfaces of natural organisms exhibit excellent wetting properties, which are especially critical in oil–water separation, surface cleaning, water repellency, and stain resistance. In nature, many plants, insects, and animals have evolved surfaces with smooth properties that help them survive in harsh environments [98]. The smooth surfaces of living organisms are widely found in the Earth’s major ecosystems, including the sky, land, and sea. Typical examples include the superhydrophobic surface of lotus leaves [99], the scales of geckos [100], the compound eyes of mosquitoes [101], the legs of water striders [102], rice leaves [103], and butterfly wings [104] (Fig. 5e–j) [105]. The smooth properties of these surfaces are mainly used for fluid manipulation to help organisms adapt to complex natural environments. As an example, the surface of lotus leaves is superhydrophobic through special microstructures that allow rainwater to roll off quickly, carrying away dust and microorganisms [106], while the gecko’s bristles enable it to crawl on vertical surfaces, utilizing microstructures to enhance adhesion [107]. These surface properties enhance the adaptability of organisms in different environments. Although these smooth surfaces are similar in principle, they differ in their liquid handling and surface behavior. These differences arise from the unique microstructure and chemical composition of the organism’s surface. The surface diversity of organisms manifests itself not only in morphological differences but also in the richness of functions and adaptability. Inspired by these natural surfaces, researchers have designed and developed wettable materials with special functions through a bionic approach [51, 105, 108–113]. Inspired by the “lotus effect”, which arises from the synergistic combination of the micro-/nanoscale hierarchical structure and low-surface-energy wax layer on lotus leaves, Liu et al. proposed a simple strategy for fabricating a superhydrophobic Mg–Li alloy surface. They first constructed a “peony flower-like” micro-/nanoscale hierarchical structure on the Mg–Li alloy surface by hydrochloric acid etching, consisting of micron-sized protrusions combined with nanosheets. Subsequently, an ultrathin perfluorosilane low-surface-energy coating was introduced onto the rough surface via a self-assembly method. In this biomimetic design, the hierarchical micro-/nanostructure traps air to form an air cushion, greatly reducing the contact area between water and the solid surface, thereby enabling the material to follow the Cassie wetting model, while the low-surface-energy coating further decreases the surface free energy (Fig. 5l) [114]. As a result, the obtained Mg–Li alloy surface exhibited a static water contact angle as high as 160° and a sliding angle below 5°, demonstrating stable superhydrophobicity, and it still maintained excellent corrosion resistance after 180 days of exposure to air. However, in more complex hypersaline marine environments, such materials based on low-surface-energy modification often face stability challenges. Nature provides another solution to this problem. Researchers have found that the surface of kelp (Saccharina japonica) can maintain stable superoleophobicity even in saturated saline solution (Fig. 5m) [115]. This outstanding salt-resistant superoleophobicity originates from the abundance of polysaccharides on the kelp surface, particularly alginate gel, which can strongly bind water molecules in high-salinity environments to form a stable hydration layer [116, 117]. Inspired by this phenomenon, Jiang et al. successfully prepared a biomimetic coating with salt-resistant superoleophobicity using calcium alginate gel [118]. This coating not only exhibited extremely low oil adhesion (< 3 μN) toward various oils, including crude oil, silicone oil, and olive oil, but also retained excellent anti-oil-fouling performance after immersion in artificial seawater for 30 days. This biomimetic design provides a new strategy for the development of marine antifouling coatings.

Bioinspired wetting materials, by mimicking the special wettability of biological surfaces, have shown great potential in the field of oil–water separation. Notably, in nature, the superoleophobicity of biological surfaces can be categorized into two types: superoleophobicity in air and underwater superoleophobicity. Surfaces that are superoleophobic in air, such as lotus leaves [123] and springtail cuticles [124], trap an air layer due to their micro-/nano-composite structures and low-surface-energy chemical components, causing oil droplets to suspend on the air layer and thus exhibiting superoleophobicity. In contrast, underwater superoleophobic surfaces, such as fish scales [125] and algae surfaces [126], form a stable “hydration layer” through their micro-/nano-rough structures, hydrophilic chemical components, and water molecules. This layer prevents the oil phase from penetrating, thereby achieving underwater superoleophobicity. These natural mechanisms provide key ideas for the design of efficient oil–water separation materials, such as the construction of micro- and nanostructures, the modulation of surface chemistry, and the introduction of air–liquid interfaces to achieve selective wetting and separation. Dong et al., inspired by the barbed structure of cat tongues and the liquid-guiding mechanism of Nepenthes pitcher lips, used 3D printing to construct a complementary meshing gear topology (Fig. 5a) [119]. Different superwetting surfaces guided oil and water to form separate liquid films, and the rotation and squeezing of the gears enabled continuous separation of oil microdroplets and emulsions, achieving a separation efficiency of 99.4% and a flux of 2000 L m^−2^ h^−1^. This dynamic dual-biomimetic design provides a novel biomimetic strategy for high-throughput, antifouling, continuous oil–water separation. Beyond dynamic separation through structural topology, Xue et al., inspired by the purple snail’s use of bubbles for drifting (Fig. 5b), developed a bubble-mediated dynamic antifouling membrane (CoP/SSM) that combines superhydrophilicity and underwater superoleophobicity and can electrocatalytically generate microbubbles to rapidly detach oil droplets (Fig. 5c) [120]. The membrane achieves a flux exceeding 11,920 L m^−2^ h^−1^ bar^−1^ for emulsions with separation efficiency over 99% and periodic voltage application restores decayed flux, enabling long-term self-cleaning. Additionally, Wang et al., inspired by the helical structure of cucumber tendrils [121], fabricated biomimetic spiral microchannels that utilize capillary forces and molecular polarity selectivity to separate various oil–water mixtures (Fig. 5d), achieving separation efficiency above 99% and a flux up to 292.5 L m^−2^ h^−1^. Integration of multi-channel arrays can increase flux nearly tenfold. In contrast, Chen et al., inspired by the micro-/nano-papillae of lotus leaves and the wettability patterns of desert beetles, constructed micro-nano-composite structures on polyurethane sponges using MOF-LDH, achieving superhydrophobic/superoleophilic properties (Fig. 5k) [122]. The sponges rapidly adsorb floating or submerged oil and continuously separate oil–water mixtures with an efficiency of 99.1%. Additionally, the synergy with flame-retardant coatings imparts excellent flame resistance, enhancing safety in emergency oil spill treatments. These biomimetic designs exemplify the three core strategies mentioned above—constructing micro-/nano-structures, regulating surface chemistry, and introducing gas–liquid interfaces—and collectively advance oil–water separation materials from static wetting to dynamic intelligent systems and from single-functionality to multifunctional integration.

The exquisite designs derived from biological surfaces not only reveal the principles by which nature achieves specialized wettability but also provide core design strategies and theoretical foundations for the artificial construction of smart-responsive materials. The first principle is the construction of hierarchical micro-/nano-structures. For example, the micrometer-scale papillae and nanometer-scale wax crystals on lotus leaf surfaces form a hierarchical structure that traps air to create an “air cushion”, placing water droplets in the Cassie–Baxter state and thereby achieving superhydrophobicity and self-cleaning. This principle has been widely applied in the fabrication of substrates for smart-responsive materials. By constructing rough structures on porous substrates, an ideal platform is provided for the modification of responsive molecules, and the high surface area amplifies molecular conformational changes, enabling sensitive wettability switching. The second principle is the regulation of interfacial chemistry. For instance, fish scales and algae surfaces utilize hydrophilic substances such as polysaccharides to form stable “hydration layers” with water molecules, creating a physical barrier at the solid–liquid interface and achieving excellent antifouling properties. This inspires the design of pH-, ion-, or gas-responsive materials, where the (de)protonation of surface functional groups or complexation with specific ions can reversibly control the “opening” and “closing” of the hydration layer, thereby enabling intelligent switching between hydrophilic/hydrophobic and oleophilic/oleophobic states [127–130]. The third principle is the introduction of dynamic response mechanisms. Just as awns bend and twist in response to humidity changes [131], this inspires the use of materials that respond to specific stimuli to achieve active control of wettability. This is the key feature that distinguishes smart-responsive materials from static biomimetic materials: by combining thermoresponsive, photosensitive, pH-sensitive, or other functional molecules with micro-/nano-structured substrates, molecular conformations or chemical properties can undergo reversible changes in response to environmental variations. These microscopic changes are amplified by surface roughness, ultimately manifesting as macroscopic intelligent wettability switching [132–135]. In summary, by deeply understanding the two fundamental principles of biological surfaces—“constructing micro-/nano-structures” and “regulating surface chemistry”—and integrating dynamic responsive molecular design, smart-responsive wettability materials can overcome the limitations of traditional materials, achieving a leap from “passive adaptation” to “active regulation” and providing entirely new strategies for addressing the complex and variable challenges of oil–water separation.

## Classification and Mechanisms of Smart-Responsive Wettability Materials

Smart-responsive wetting materials are a class of functional materials that can dynamically respond to external stimuli (such as temperature, light, pH, electric fields, etc.). From the perspective of wettability theoretical models, the common design principles for regulating surface wettability under different stimuli can be summarized into three levels. First, responsive functional molecules undergo reversible conformational changes or chemical reactions under external stimuli, dynamically modulating the surface free energy and altering the intrinsic contact angle (*θ*_*Y*_) in Young’s equation [38], thereby laying the molecular-level foundation for wettability switching. For example, pH-responsive materials regulate surface hydrophilicity or hydrophobicity via (de)protonation of carboxyl or amine groups. Second, pre-constructed micro-/nano-rough structures are used to amplify changes in the wetting state—according to the Wenzel [40] and Cassie–Baxter models [41], the high surface area and roughness factor (*r*) of the substrate significantly magnify molecular-level chemical signals, enabling switching from hydrophilic to hydrophobic, or even superhydrophobic states. For instance, composites of MOFs such as ZIF-8 with PNIPAM exploit the nanoscale roughness of the MOF to further amplify the conformational changes of the thermoresponsive polymer. Finally, by controlling the surface free energy through responsive molecules and combining this with the amplification effect of micro-/nano-structures on the contact angle, smart-responsive materials can achieve reversible, large-range tuning of the contact angle for target liquids. This allows intelligent switching of the capillary intrusion pressure (*ΔP*_*C*_) [50] between positive and negative values, enabling on-demand transition from an “oil removal” mode to a “water removal” mode. In summary, it is based on this set of common design principles that multi-responsive wettability materials can overcome the limitations of single-function systems, providing a flexible and universal strategy to address the complex and variable challenges of oil–water separation.

### Thermo-Responsive Smart Materials

Thermally responsive smart materials have shown promising applications in the field of water treatment and have attracted the attention of many researchers. Currently, thermo-responsive polymers such as poly(N-isopropylacrylamide) (PNIPAM), poly(vinyl alcohol-acetal), poly(N-vinyl caprolactam), etc. have been used to design and fabricate these materials [136, 137]. By combining a rough surface structure, appropriate pore size, and thermally responsive substances, these materials can effectively separate oil–water mixtures at different temperatures and even handle emulsions. The core principle of thermally responsive smart materials lies in their ability to transition between hydrophilic and hydrophobic properties at different temperatures. Temperature changes trigger adjustments in their molecular structure, which in turn change the surface energy and wettability, enabling the separation of oil and water. Such materials typically have reversible thermal sensitivity, whereby the surface can shift between hydrophobicity and hydrophilicity when the temperature is raised or lowered. Common mechanisms include the following: (1) Surface-modified polymers: e.g., PNIPAM, which exhibit hydrophilicity below the critical dissolution temperature and become hydrophobic above that temperature. (2) Liquid crystal phase transition: certain liquid crystal materials undergo structural changes with temperature changes, altering the surface energy and realizing selective adsorption of oil or water. (3) Phase separation effect: Under temperature change, microscopic phase separation occurs in the internal structure of the material, which contributes to the separation effect of oil and water [138–141]. This diversified thermal response mechanism enables the smart materials to exhibit excellent oil–water separation performance under different application conditions, which promotes their wide application in water treatment and environmental protection.

Based on the diverse thermo-responsive mechanisms mentioned above, Feng et al. prepared a smart separation membrane with switchable wettability by modifying a nylon microfiltration membrane with the temperature-sensitive polymer PNIPAM [142]. When the temperature is below its LCST (25 °C), the PNIPAM molecular chains extend due to intermolecular hydrogen bonding with water, rendering the membrane hydrophilic/underwater superoleophobic. When the temperature is above the LCST (45 °C), the molecular chains collapse to form intramolecular hydrogen bonds, exposing hydrophobic isopropyl groups and transitioning the membrane to a hydrophobic/superoleophilic state. Consequently, the membrane can efficiently separate various oil-in-water emulsions (including cationic, non-ionic, and anionic types) at 25 °C and separate water-in-oil emulsions at 45 °C. The separation efficiency for at least 16 stable emulsions exceeds 97.8%, and performance remains nearly unchanged after 10 temperature cycles. This research provides a simple and low-cost smart membrane material solution for on-demand treatment of complex oily wastewater. Huang et al., on the other hand, grafted PNIPAM onto a carbon nanotube/chitosan aerogel skeleton [143]. Building upon the same temperature-sensitive mechanism, they introduced highly thermally conductive carbon nanotubes to construct a three-dimensional porous network, significantly enhancing the thermal response rate and adsorption capacity. This aerogel can rapidly adsorb various oils from water at 45 °C (up to 53 times its own weight) and requires only 15 min in 25 °C water for desorption. Its performance remains nearly unchanged after 15 cycles, offering an efficient and recyclable smart material solution for temperature-controlled oil–water separation. Yuan et al. further took the approach by growing CoZn-ZIF in situ on PVDF/PNIPAM electrospun membranes [144]. Utilizing the thermo-sensitive conformational changes of PNIPAM combined with the micro-/nano-roughness provided by ZIF, they achieved reversible switching between superhydrophilic/underwater superoleophobic and superhydrophobic/oleophilic-under-water states. This enables efficient separation of oil-in-water and water-in-oil emulsions at 25 and 50 °C, respectively (flux > 1900 and 1150 L m^−2^ h^−1^, efficiency > 99.0% and 99.6%). More importantly, the CoZn-ZIF endows the membrane with catalytic degradation functionality, capable of degrading 99.92% of methylene blue within 15 min via peroxymonosulfate (PMS) activation. This integrates temperature-controlled emulsion separation with organic pollutant degradation, achieving a multifunctional expansion from single-stage separation to synergistic separation degradation.

In summary, the preparation techniques for thermo-responsive materials are relatively mature, enabling large-scale production through methods such as electrospinning and surface grafting. Polymers like PNIPAM are chemically stable in neutral environments, but prolonged exposure to high temperatures may lead to chain degradation. Cross-linking modification can effectively enhance their mechanical stability, resisting structural fatigue caused by repeated contraction/swelling cycles, thereby achieving hundreds of stable cycles without significant performance decay [145]. The preparation process for these materials does not require toxic solvents, and biodegradable polymers can be selected to reduce environmental burden [146]. They are particularly suitable for scenarios with temperature fluctuations, such as industrial wastewater cooling processes or geothermal areas. However, their response speed is limited by heat transfer efficiency, and issues with uneven temperature distribution may arise in large-volume systems.

### PH-responsive Smart Materials

Thermally responsive smart materials provide efficient solutions for oil–water separation in water treatment by regulating hydrophilicity and hydrophobicity through temperature; however, complex water environments are often characterized by multiple changes in physical and chemical properties, and a single temperature-responsive mechanism is difficult to satisfy the needs of all application scenarios. Therefore, pH-responsive smart materials, which are complementary to thermally responsive mechanisms, have attracted a lot of attention due to their high sensitivity to the chemical environment. Similar to thermally responsive smart materials, pH-responsive smart materials have become very popular in the research and development field due to their ease of handling and fast response time. pH-responsive smart materials refer to a class of functional polymer materials whose physical or chemical properties (e.g., volume, shape, hydrophilicity, permeability, and solubility, etc.) are able to undergo a reversible and significant change in response to changes in the pH value of the environment [147]. These materials can “sense” the changes in pH of the surrounding environment and make corresponding “response,” showing intelligent behavior. The core principle is based on the fact that the molecular chain of the material contains a large number of ionizable or protonatable/deprotonatable functional groups (i.e., weak acid or weak base groups). These groups ionize under different pH environments, leading to changes in the charge, conformation, and interchain forces of the molecular chains and thus to changes in macroscopic properties. These materials usually consist of polymers (e.g., polyvinyl alcohol and polyurethane, etc.) with pH-responsive functional materials (e.g., microcapsules and nanoparticles, etc.) that are able to exhibit different physical or chemical properties in response to pH changes. The structure of these materials contains groups that react with H^+^ or OH^−^ ions, and the process of ionization or deionization leads to changes in the hydrophilic and hydrophobic properties of the materials [148–152]. For instance, certain groups enhance hydrophilicity in acidic environments, while they may exhibit hydrophobicity in alkaline environments for applications such as oil–water separation.

**Fig. 6:**
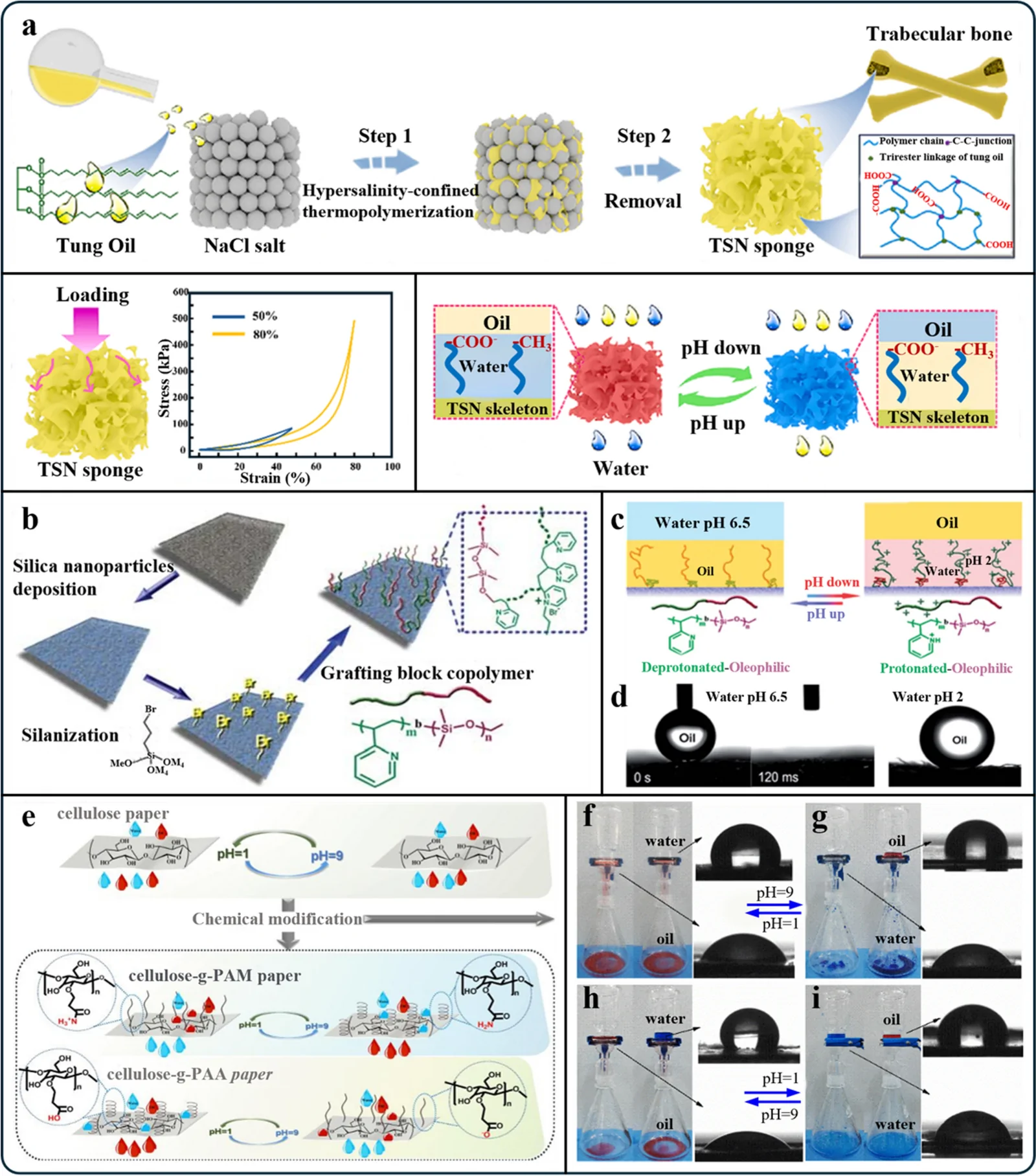
**a** Schematic of the preparation process of TSN sponge with smart pH responsiveness and superelasticity. Reproduced from Ref. [153] with permission, copyright 2024, Elsevier BV. **b** Schematic representation of the preparation of superoleophilic and superoleophobic switchable surfaces on nonwoven fabrics. **c** Schematic diagram of switchable oil wettability of P2VP-b-PDMS-grafted fabrics. Left, schematic of oil wettability of functionalized textiles in water at pH 6.5. Right, schematic diagram of oil wettability of functionalized textiles in water with pH 2.0. **d** Wettability of P2VP-b-PDMS-coated fabrics in aqueous media of different pH. At pH 6.5, DCE droplets are immediately drawn into the fabric within 0.12 s, exhibiting superoleophilic properties. pH = 2.0, DCE droplets form spherical shapes on the surface, exhibiting superoleophobic properties. Reproduced from Ref. [154] with permission, copyright 2012, Springer Science and Business Media LLC. **e** Preparation of cellulose-g-PAA and cellulose-g-PAM papers by chemical modification. The pH response of cellulose-g-PAA paper (**f, g**) and cellulose-g-PAM paper (**h, i**) to oil–water separation and contact angle. Reproduced from Ref. [155] with permission, copyright 2019, Elsevier B.V

Typical pH-responsive functional substances include pyridine, carboxyl, acrylic, acrylamide, and tertiary amines. These groups not only impart responsive properties to the materials, but also influence its application. By combining these functional substances with porous materials (e.g., metal mesh, polymer mesh, and foam, etc.), many pH-responsive smart materials with excellent properties have been prepared. In recent years, pH-stimulation-responsive biomass sponges have emerged as potential materials for oily wastewater purification due to their rapid response, gentle operation, and wettability switching without additional energy consumption. Liu et al. designed a tung oil-derived sponge material (TSN) inspired by bone trabeculae structure, which was prepared by a high-salt-limited domain thermal polymerization strategy (Fig. 6a) with smart pH-responsive wettability and superelasticity (maintaining 100% strain rate and height after 1000 compression cycles). Analyzed from the wettability theoretical model, the efficient separation performance of the sponge is closely related to the modulation of the surface wetting state. In the hydrophobic/lipophilic state (pH = 7), the oil phase exhibits a fully infiltrated rough structure (*θ*_*W*_ < 90°) as modeled by Wenzel [40]. According to the Wenzel equation, the high roughness (*r* > 1) amplifies the lipophilicity of the material, allowing the oil phase to rapidly penetrate the pores and ensuring a high flux (6700 L m^−2^ h^−1^). When a change in the pH environment triggered the material to shift to a hydrophilic/oleophobic state (pH = 13), a hydrated layer was formed on the surface and the oil phase presented the Cassie–Baxter model [41] of a composite solid–liquid–liquid interface (underwater oil contact angle of 137.1°). The reduction of contact angle hysteresis [45] endowed the material with fouling resistance, while the positive oil-phase intrusion pressure *ΔP*_*C*_ [50] was able to stably keep the oil phase out of the material, ensuring separation stability (nearly 100% performance retention for 1000 cycles). The sponge can efficiently separate a variety of oil–water mixtures and emulsions (separation efficiency > 99.4%) and adapt to harsh environments, providing a new idea for the design of renewable vegetable oil-based smart adsorption and separation materials [153]. However, its wettability switching rate is affected by the sponge pore structure, which still needs to be optimized for complex emulsion separation stabilized by surfactants. In contrast, Zhang et al. developed a smart surface based on pH-responsive block copolymer (P2VP-b-PDMS) grafting, which can realize reversible switching of superoleophilicity and superoleophobicity in aqueous media (Fig. 6b). The surface controls the degree of exposure of the oleophobic PDMS chains by altering the conformation through protonation/deprotonation of the P2VP chains, thus enabling selective penetration or repulsion of oil at different pH (Fig. 6c, d). Functionalized textiles and polyurethane sponges can be used for controlled oil–water separation, such as selective filtration of the oil or water phase, and for reversible capture and release of underwater oil, providing new strategies for smart separation material design [154]. However, it relies on synthetic polymer block copolymers, and the preparation process involves multi-step modifications such as silylation, which is less biomass-compatible and more costly. In contrast, He et al. prepared two biomass cellulosic materials (cellulose-g-PAA and cellulose-g-PAM) with opposite pH responsiveness by grafting acrylic acid and acrylamide (AM) onto eucalyptus pulp cellulose, respectively (Fig. 6e). These two materials reversibly switched surface wettability under different pH conditions (pH = 1 and 9): cellulose-g-PAA was hydrophobic/lipophilic under acidity and hydrophilic/lipophobic under alkalinity, whereas the opposite was true for cellulose-g-PAM. The response mechanism stems from protonation/deprotonation of carboxyl and amide groups and conformational changes of the molecular chain (Fig. 6f–i) [155]. The materials exhibit high selectivity in oil–water separation (efficiency > 97%) and can be pH-regulated for oil adsorption and desorption, providing a green and degradable solution for smart separation materials.

However, its function is limited to physical separation and lacks the ability to treat soluble organic pollutants. In comparison, the HATP@CN composite membrane constructed by Cai et al. introduced TiO_2_ photocatalytic function on the basis of pH-responsive separation, which not only realized pH-regulated high-throughput oil–water separation (flux > 6800 L m^−2^ h^−1^), it also enables rapid degradation of organic dyes under UV light (> 90% in 6 min) by reactive oxygen species such as –OH and –O_2_^−^ produced by TiO_2_ [156]. The catalytic principle is that TiO_2_ is excited by light to generate electron–hole pairs, which in turn generates strong oxidizing free radicals to mineralize dyes into CO_2_ and H_2_O. What’s more, pH-responsive wettability switching on the surface of the membrane can modulate the contact efficiency of dye molecules with the active sites of TiO_2_ to achieve synergistic separation and degradation, which provides a multifunctional and integrated solution for the treatment of complex oily wastewater. Although Cai et al. extended the functional boundaries of pH-responsive materials by introducing photocatalysis, these advances are still limited to the laboratory pilot scale. To achieve industrial deployment, engineering challenges such as scale-up preparation, material regeneration, and secondary contamination control need to be addressed. Tang et al. accordingly proposed an engineering-oriented ectopic preparation strategy by first synthesizing pH-responsive magnetic core–shell nanoparticles (Fe_3_O_4_@SiO_2_), which were then loaded onto sponges, metal meshes, and other substrates by dip coating [157]. The convenient preparation of large-size materials was realized, breaking the bottleneck of in situ methods in size scaling up. The whole process of “oil absorption–desorption-particle recovery-secondary separation” is designed: pH-responsive sponge treats the oily wastewater and regenerates it in situ, magnet recovers the dislodged particles (recovery rate of 88%), and pH-responsive metal mesh treats the secondary oily wastewater (three-phase mixture of oil–water-oil). Based on the above research, the preparation methods for pH-responsive materials are diverse, achievable through techniques such as dip coating, layer-by-layer self-assembly, and graft polymerization [158]. They offer a wide range of substrate options and are amenable to scale-up production. Polyacids/polybases are chemically stable within the pH 2–10 range, but may hydrolyze under extreme pH conditions. Cross-linking treatments can enhance their mechanical stability, resisting volume changes caused by repeated protonation/deprotonation cycles and ensuring a high wettability recovery rate even after dozens of cycles [159]. The preparation process typically avoids organic solvents, and the acid/base solutions used can be recycled. This makes them particularly suitable for applications in acid/base wastewater treatment, chemical industrial park wastewater, and the biomedical field. Compared to thermo-responsive materials, pH-responsive materials offer lower energy consumption, but they require a continuous supply of acid or base solutions, and the energy consumption associated with waste liquid treatment must be considered.

### Photo-Responsive Smart Materials

Thermal-responsive and pH-responsive smart materials provide an effective solution for the separation of complex oil–water systems through the perception and feedback of the external temperature and chemical environment; however, whether relying on thermal triggering or pH change, the response process is still limited by the physical or chemical conditions of the environmental medium itself, which to some extent restricts the precision of control and the applicable scenarios. In order to further realize the remote, non-contact, high temporal and spatial precision active control of the separation process, a new class of light-responsive smart materials has emerged, which inherits the core design concept of “intelligent switching of wettability” of environment-responsive materials, and at the same time introduces light as a more advantageous external control signal. Similar to pH-responsive materials, light-responsive smart materials are attracting attention for their highly efficient environmental response properties. Light-responsive smart materials for oil–water separation are a class of functional materials or coatings whose surface wettability (hydrophilicity/hydrophobicity) can be reversibly or irreversibly switched by light irradiation at a specific wavelength [160]. Through remote and precise manipulation like light irradiation, on-demand and highly efficient separation of a wide range of oil–water mixtures (including emulsions that are difficult to separate) can be realized, and the switching of separation modes can be accomplished in a single set of devices [161]. The core principle is that the photosensitive molecules on the surface of the material undergo physical or chemical changes after absorbing photons, thereby altering the surface free energy and microstructure and ultimately realizing the intelligent conversion of wettability.

**Fig. 7:**
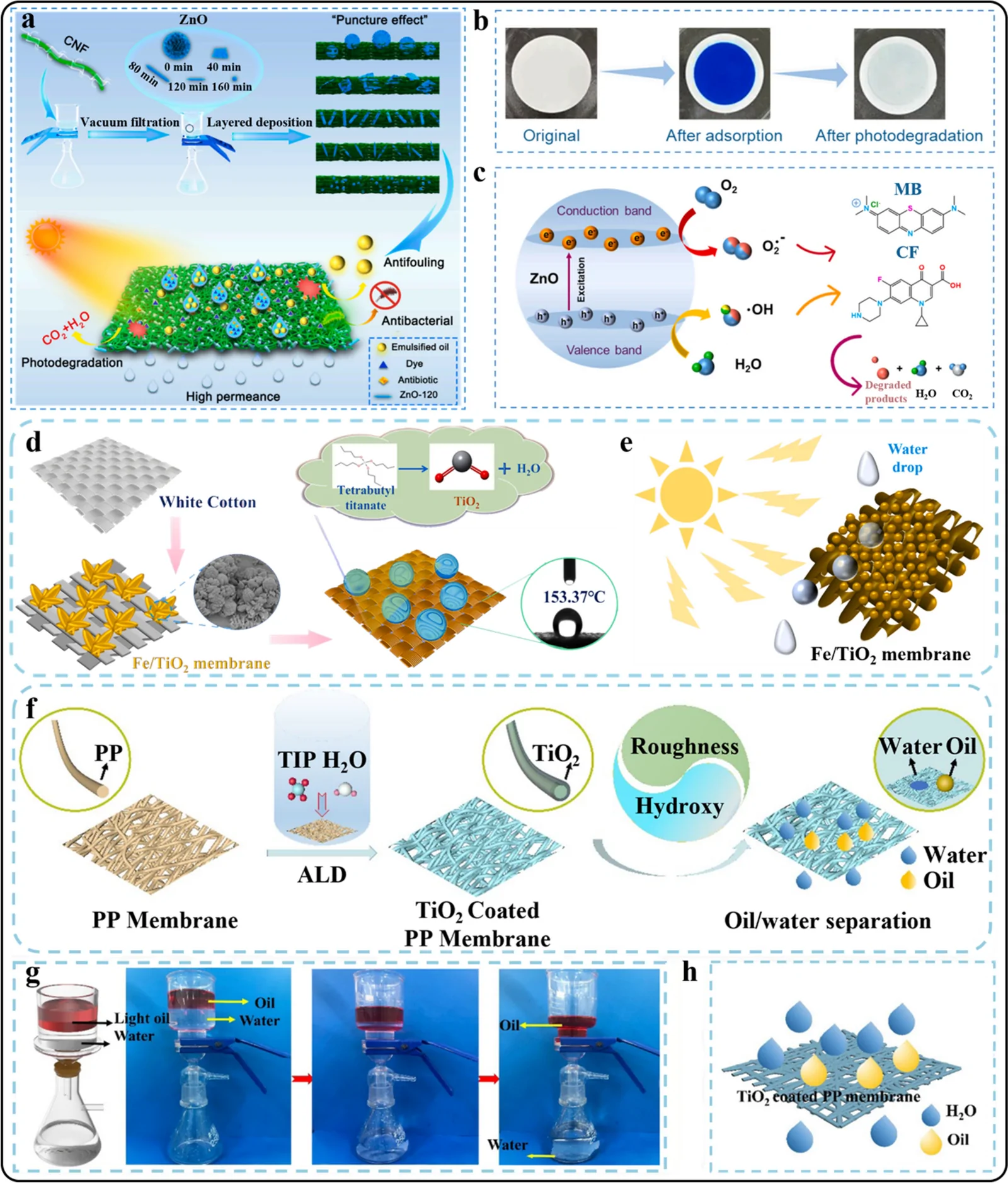
**a** Schematic of the fabrication process of CM-ZnO. **b** Photographs of CM-ZnO-120 before and after photodegradation. **c** illustrating the photocatalytic degradation of MB and CF. Reproduced from Ref. [166] with permission, copyright 2024, Elsevier BV. **d** Schematic diagram of Fe/TiO_2_ membrane preparation process. **e** Superhydrophobic diagram of the Fe/TiO_2_ membrane after illumination. Reproduced from Ref. [167] with permission, copyright 2024, Elsevier BV. **f** Schematic diagram of ALD-built TiO_2_-coated PPM and oil–water separation process. Separation of oil–water mixtures by PPM-ALD-200: **g** Light oil (petroleum ether/water) mixture. **h** Schematic diagram of the “water-removing” oil–water separation membrane. Reproduced from Ref. [168] with permission, copyright 2021, Springer Science and Business Media LLC

Typically, ultraviolet (UV) or visible light induces a reversible shift in surface wettability, which enables on-demand and efficient separation of oil–water mixtures. In recent years, photo-responsive nanoparticles such as TiO_2_ [162], zinc oxide (ZnO), and tin dioxide (SnO_2_) have been widely explored and applied, which exhibit switchable superhydrophilic properties under UV light or dark conditions [163–165]. In addition, photocatalytic degradation is considered to be one of the most effective remediation strategies for oily wastewater. Lou et al. prepared a hybrid ultrafiltration membrane based on nanocellulose and zinc oxide nanoparticles (ZnO-NPs) by a simple layered filtration method without chemical modification (Fig. 7a). The membrane combines the high specific surface area of nanocellulose and the “puncture effect” of ZnO-NPs to form abundant nanochannels in the membrane, which significantly improves the permeability of the membrane and exhibits excellent water permeability. The introduction of ZnO-NPs not only improves the structure of the membrane, but also significantly increases its wet tensile strength, enhancing its stability and durability in the wet state. The membranes are highly efficient in separating nanoemulsions through size exclusion and emulsion breaking and have excellent water–oil separation capability. What’s more, CM-ZnO-120 prepared in this paper has the potential to act as a potent and long-lasting photocatalyst for degrading organic pollutants in wastewater (Fig. 7b, c) [166]. However, focusing on oil-phase separation and photothermal conversion, Gao et al. prepared a superhydrophobic Fe/TiO_2_ membrane obtained by dynamic etching with iron salts, which was based on cotton cloth and dynamically etched with titanium dioxide microspheres using stainless steel mesh in an acidic environment to form a “petal-like” micronanostick structure with good hydrophobicity and lipophilicity (Fig. 7d). The water contact angle in air reaches 140.16°, the average oil flux reaches 16,221.88 L m^−2^ h^−1^, and the O/W emulsion can be separated effectively. Meanwhile, Fe doping extends the light absorption range of TiO_2_ to the visible region, and the surface temperature of the membrane can be increased to 50 °C in 2 min under simulated sunlight, which has the ability of photothermal conversion (Fig. 7e) [167]. The hydrophobicity of the membrane was enhanced after illumination, and the average separation efficiency of the four oils was increased to 96.81%, with a flux of 18,154.67 L m^−2^ h^−1^, which provides an environmentally friendly and sustainable solution for the treatment of oily wastewater and the degradation of organic dyes. Li et al. prepared superhydrophilic and underwater superoleophobic polypropylene (PP) membranes by atomic layer deposition (ALD) for gravity-driven high-efficiency oil–water separation (Fig. 7f). Due to the synergistic effect, the TiO_2_-coated PP membranes can exhibit excellent oil resistance underwater with an oil contact angle of more than 150°. The superwettability of such membranes enables easy separation of oil–water mixtures with separation efficiencies of more than 95% (Fig. 7g, h) [168]. In addition, the TiO_2_-coated PP membranes have good recyclability and mechanical stability, showing great promise for practical oil–water separation applications.

In summary, the scalability of preparation methods for photo-responsive materials depends on the specific system: inorganic oxides like TiO_2_ can be prepared on a large scale via sol–gel or hydrothermal methods, but controlling their morphology poses significant challenges; organic molecules like azobenzene involve complex synthesis steps, making large-scale production difficult [169]. It is worth noting that although azobenzene can undergo multiple photo-isomerization cycles, long-term light exposure may lead to molecular fatigue [170]. In contrast, inorganic materials such as TiO_2_ exhibit excellent resistance to acid and alkali corrosion. Their adhesion can be enhanced through methods like atomic layer deposition, ensuring stable operation during cycles of UV excitation and dark recovery. The photocatalytic properties of TiO_2_ also enable simultaneous degradation of pollutants, making it particularly suitable for treating oily or dye-contaminated wastewater, as well as for applications in outdoor facilities and marine environments [171]. Compared with pH-responsive materials, photo-responsive materials enable remote, non-contact control with more precise response. However, their light penetration depth is limited, potentially leading to uneven response in thick samples.

### Electric-Responsive Smart Materials

While the photo-responsive properties of smart materials provide a new approach to efficient and controllable oil–water separation, electrically responsive technologies show unique advantages for more precise dynamic regulation of wettability. Photo-responsive materials rely on semiconductors or photochromic molecules to change their properties. In contrast, electric wetting technology directly regulates the solid–liquid interfacial energy by applying an external electric field. This approach enables instant and reversible switching of wettability. When a voltage is applied between a liquid droplet and the conductive solid substrate below it, an electric charge accumulates on both the liquid side and the solid electrode, which leads to a decrease in the interfacial energy and consequently a change in wettability from hydrophobic to hydrophilic [17, 172]. This fast response mechanism, based on the electrostatic effect, complements well the properties of photo-responsive materials. The two technologies have their own strengths: photo-responsive has the advantage of wireless remote regulation, while electro-wetting enables more precise real-time control, and their synergistic application offers more possibilities for developing a new generation of smart separation materials.

**Fig. 8:**
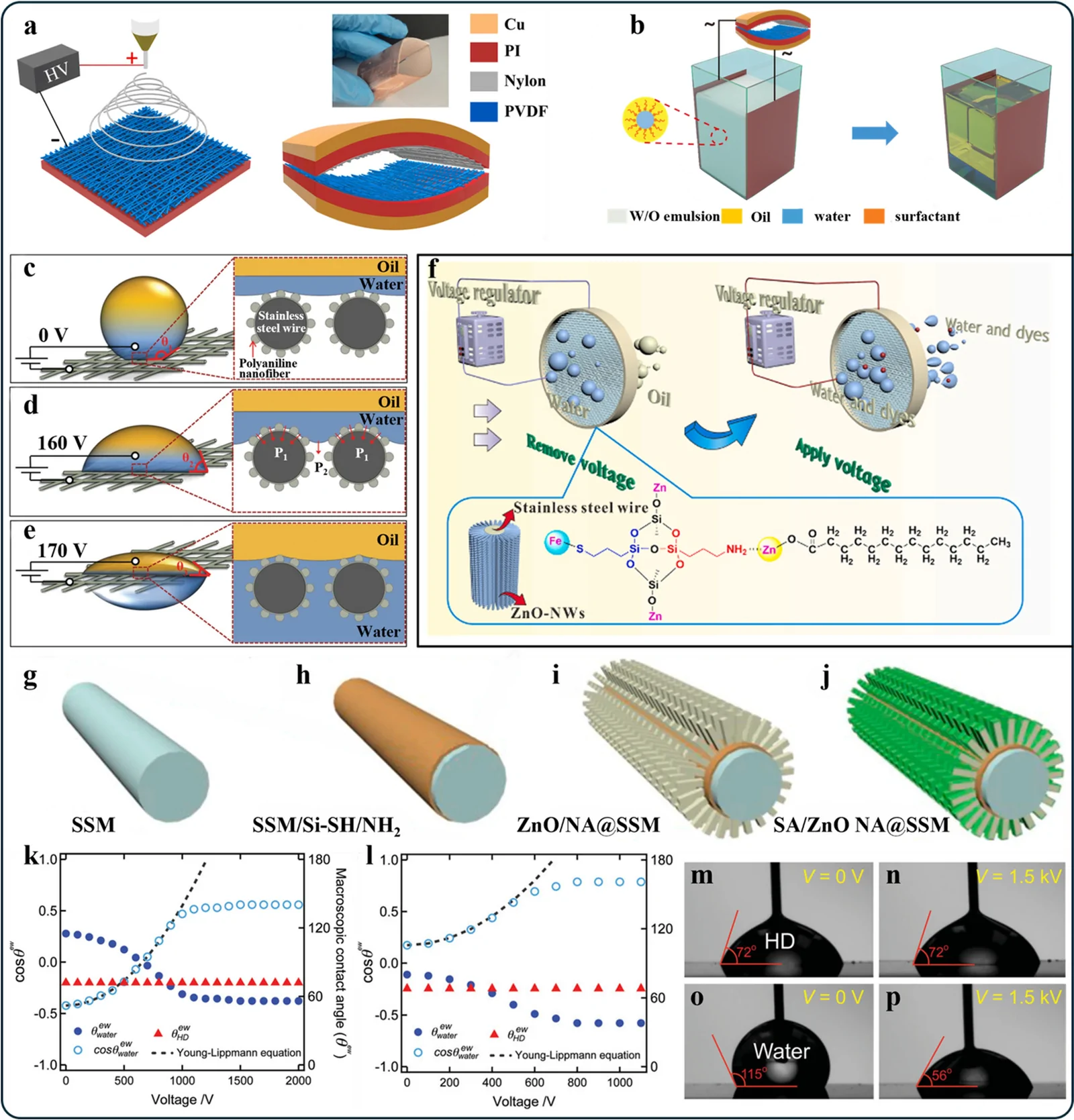
**a** Schematic of preparation of electrospun nylon and PVDF nanofibers, **b** structure of electrospun nylon and PVDF nanofibers-based triboelectric nanogenerator (ENTENG) (the inset is the front view of the fabricated ENTENG), Reproduced from Ref. [173] with permission, copyright 2021, Elsevier BV. **c** Schematic diagram of the electric field-induced oil–water separation process based on the polyaniline network structure. A small amount of water is added to the device to ensure that the oil–water mixture does not penetrate the mesh membrane. Without an applied voltage, the water is difficult to wet the superhydrophobic mesh structure with a contact angle≈146°. At the same time, due to the superhydrophobic properties, the oil will stay on top of the mesh, so both water and oil will stay on top of the mesh. **d** When an applied voltage reaches 160 V, the water–air interface sags, water will wet the mesh to some extent by increasing the ECP, and oil will remain on the mesh. **e** When a voltage of 170 V is applied, the water on the mesh transforms to the Wenzel state, with a water contact angle of about 40°, and penetrates into the mesh, while the oil remains above the mesh due to its superhydrophobicity under water. The corresponding enlarged schematic diagrams of liquid–solid contact are shown on the right. The results show that as the electric field strength increases, water gradually penetrates into the grid, and the macroscopic water contact angle (*θ*) decreases from =146° to =40°. Even though water penetrates into the grid, oil cannot wet the grid because it is stably superhydrophobic underwater. Reproduced from Ref. [174] with permission, copyright 2016, Wiley. **f** Preparation: an improved process was used to form ZnO-NAs in situ on a modified SSM surface (SA/ZnO NA@SSM) with high interfacial bond strength, while also improving durability. The water wettability of the lattice can be adjusted with or without the application of an electric field. Large-area views of sample images: **j** original grid, **h** SSM/Si-SH/NH_2_, **i** ZnO NA@SSM, and **j** SA/ZnO NA@SSM. Reproduced from Ref. [175] with permission, copyright 2021, Elsevier BV. **k** Macroscopic contact angle of water and hexadecane (HD) as a function of voltage applied to the non-textured substrate. **l** Macroscopic contact angle of water and hexadecane (HD) in the presence of PS80 as a function of voltage applied to the non-textured substrate. **m, n** The macroscopic contact angle for hexadecane remains unchanged with increasing voltage. **o, p** The macroscopic contact angle for water decreases with increasing voltage. Reproduced from Ref. [176] with permission, copyright 2012, Wiley

Oily wastewater poses a serious threat to the ecological environment and human health. Especially, water mixed in lubricating oil will accelerate the corrosion and wear of mechanical equipment and even lead to mechanical failure. Wang et al. designed a TENG based on nylon and PVDF electrospun nanofibers (Fig. 8a), the core function of which is to generate an asymmetric alternating current (AC) electric field by friction and to achieve efficient separation of W/O emulsions by utilizing the electrical response property (Fig. 8b) [173]. A voltage of up to 2847 V and a short-circuit current of 115 μA can be output through a bis-polyimide (PI) film as a charge storage transition layer. The water content of the emulsion was reduced from 5 to 0.15 wt% in 30 min at 2500 V, resulting in a separation rate of 96.97%. The mechanism is a synergistic effect of dipole aggregation, oscillatory aggregation, and electrophoretic aggregation, and wind energy can be utilized to drive a large amount of emulsion separation, which has the advantages of high efficiency, low cost, and safety and has the potential to be applied in the field of industrial lubricant treatment [177]. While this TENG-based system excels in handling stable W/O emulsions and achieving self-powered separation with natural energy, its high working voltage (2500 V) and relatively complex device structure may limit its application in scenarios requiring simple operation or low-voltage input. In contrast, Tian et al. prepared nanostructured polyaniline on a microscale stainless steel mesh using emulsion polymerization. This micro-/nanoscale hierarchical structured polyaniline mesh has stable underwater superoleophobicity and smartly switchable superwettability, where superhydrophobicity becomes hydrophilicity at 160 V, and water can penetrate the mesh at 170 V, while oil stays on the mesh **(**Fig. 8c–e) [174]. In addition, the mesh has low underwater oil sticking ability and corrosion resistance, which can keep working in harsh environments in practical applications. However, its relatively higher response voltage (160–170 V) and reliance on emulsion polymerization for fabrication may limit its efficiency in scenarios requiring rapid, low-energy operation. Moreover, Wang et al. reported a smart stainless steel mesh (SA/ZnO NA@SSM) (Fig. 8g–j) [175] with reversible superhydrophobic–hydrophilic properties for electrically induced oil–water separation prepared by a two-step surface modification and hydrothermal method (Fig. 8f). The material can realize the wettability transition within seconds at low voltage (15 V), which is more efficient and safer compared to the high voltages (e.g., 2 kV) required in previous studies. The reversible transition between the superhydrophobic state (contact angle 159°) and the hydrophilic state (contact angle 40°) is realized by electric field control and is suitable for both “oil-removing” and “water-removing” modes of separation, with efficiencies of up to 99%. However, its application is mainly focused on simple oil–water mixtures and relies on hydrothermal preparation, which may limit scalability for large-volume emulsion separation. In contrast, Tuteja et al. prepared a filtration membrane of 50 wt% fluorodecyl POSS mixed with x-PDMS capable of flexibly separating various oil–water mixtures by applying an electric field. The study tested and compared the electrowetting behavior of water and hexadecane on this substrate and showed that the contact angle of hexadecane (contact angle = 72°) was independent of voltage, whereas the contact angle of water decreased from 115° at 0 kV to 56° at 1.5 kV. This change in wettability indicates a significant difference in the wetting behavior of oil and water under the action of an electric field (Fig. 8m–p) [176]. The method utilizes the principle of electro-wetting to construct an oleophobic membrane with a specific texture with the help of a nylon membrane co-coated with fluorinated POSS and PDMS blends, and the wettability of the polar liquid (water) is modulated by applying an electric field to change from the Cassie–Baxter state to the Wenzel state and permeate the membrane, whereas the nonpolar liquid (oil) is retained (Fig. 8k–l). The separation efficiency of the method was shown to be ≥ 99.9% for free oil–water mixtures, W/O emulsions, and O/W emulsions. In addition, the device is easy to be scaled up, and the designed continuous separation device can continuously process emulsions with an aqueous phase flux up to 200 L m^−2^ h^−1^, which is promising for a wide range of applications in the fields of oil spill cleanup, fuel purification, and wastewater treatment.

Based on the above discussion, the scalability of preparation methods for electro-responsive materials varies: conductive polymers can be prepared via electrochemical polymerization, but achieving uniform coating over large areas still presents technical challenges [178]; whereas composite membranes based on metal mesh substrates have shown good potential for scale-up. Conductive polymers like polyaniline are relatively chemically stable during the doping process, but long-term electrochemical cycling may lead to material degradation. Metal mesh substrates inherently possess high mechanical strength, and stable interfacial bonding can ensure reliable operation over hundreds of cycles [179]. However, the use of fluorinated polymers may pose environmental risks. These materials are suitable for applications such as dewatering low water content oil, electro-responsive smart switches, and wearable devices [180]. Compared with photo-responsive materials, electro-responsive materials offer faster response speeds and higher control precision, but they require electrode configuration and conductive substrates, demanding a higher level of system integration.

### Gas-Responsive Smart Materials

**Fig. 9:**
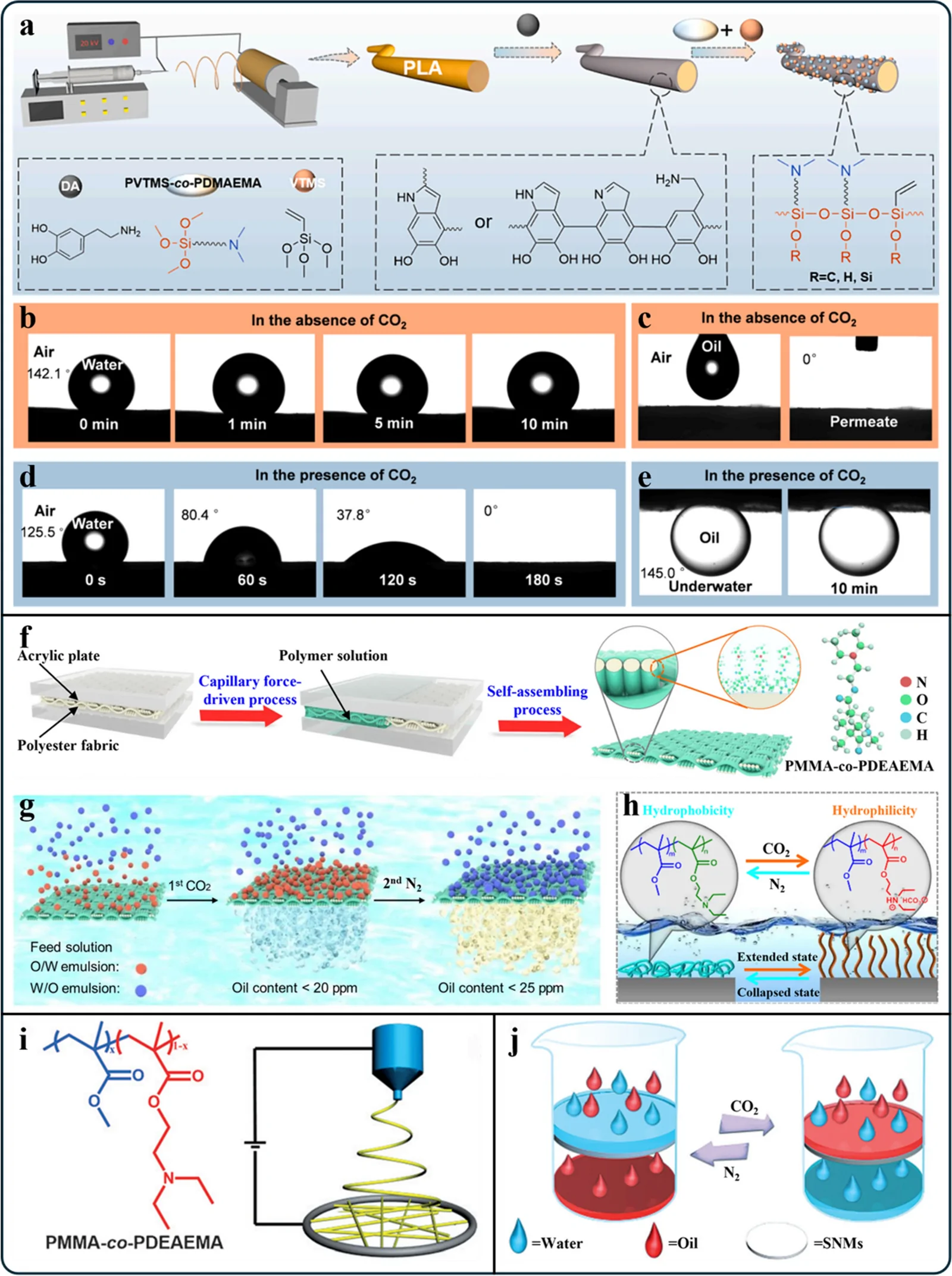
**a** Preparation scheme of PLA@PDA/DMA-V NFMs. **b** Water and **c** oil wetting behavior of PLA@PDA/DMA-V NFMs surface under CO_2_ stimulation in air. **d** Wetting behavior of water in air and **e** wetting behavior of oil underwater on PLA@PDA/DMA-V NFMs surface under CO_2_ stimulation. Reproduced from Ref. [184] with permission, copyright 2024, Elsevier BV. **f** Schematic of the process for the preparation of PPFM by capillary force-driven constrained self-assembly method. The gas switches the separation and self-cleaning properties of the multiphase emulsion mixture. **g** Schematic of the two-step separation process of a multiphase emulsion system under CO_2_/N_2_ stimulation. **h** Schematic of the surface wetting mechanism of PPFM under CO_2_/N_2_ stimulation. Reproduced from Ref. [185] with permission, copyright 2023, Springer Science and Business Media LLC. **i** Preparation process of smart nanofilms and CO_2_-switchable oil–water wettability. Schematic of the process of preparing PMMA-co-PDEAEMA nanofibers by electrostatic spinning. **j** Schematic illustration of CO_2_-switchable oil–water switching. Reproduced from Ref. [186] with permission, copyright 2015, Wiley

The wettability regulation technology of smart materials is developing in a diversified direction, from optical/electrical response to gas response, forming a multi-mode synergistic smart regulation system. While photoelectric synergistic regulation shows unique advantages, gas-responsive smart materials offer another environmentally friendly solution for oil–water separation. Unlike photoelectric regulation, which requires external field intervention, gas-responsive materials can be triggered by environmentally friendly gases, such as carbon dioxide, to realize a reversible shift in surface wettability. Specifically, the surface wettability (hydrophilicity/hydrophobicity) can be significantly and reversibly changed when exposed to a specific gas or atmosphere [181]. The “on/off” regulation of surface properties can be achieved by simply switching the gas (e.g., CO_2_, O_2_, air, hydrogen, or organic vapors), which is an intelligent response strategy that is easy to operate, consumes little energy, and is usually residue-free. The core principle involves functional molecules on the material surface undergoing reversible reactions or physical interactions with specific gas molecules. These interactions alter molecular conformation, surface energy, or microstructure, leading to a macroscopic switch in wettability. In addition, gases such as NH_3_, O_2_, and H_2_ are used to modulate the surface properties of the materials to support precise and smart oil–water separation [182, 183]. With further research, such materials are expected to be widely used in industrial wastewater treatment, environmental treatment and oil, and gas recovery to improve resource utilization efficiency and reduce environmental pollution. Wang et al. opened a PLA nanofiber membrane (PLA@PDA/DMA-V NFMs) with switchable CO_2_-responsive wettability, which was prepared by grafting CO_2_-responsive copolymers onto PLA nanofibers with the assistance of a polydopamine (PDA) coating (Fig. 9a). The membranes can realize reversible transition between hydrophobic/lipophilic and hydrophilic/lipophobic under alternating CO_2_/N_2_ stimulation and thus have the function of “oil-absorbing” or “water-absorbing” on demand (Fig. 9b–f). The high flux (up to 5015 L m^−2^ h^−1^), high separation efficiency (> 99.83%), and good recyclability for a variety of immiscible oil–water mixtures and emulsions provide a new strategy for the development of energy-saving and environmentally friendly oil–water separation materials [184]. However, the scale-up preparation is limited by the electrospinning process and is less targeted for the separation of multiphase complex systems. In contrast, Dong et al. designed a large-area (up to 3600 cm^2^), scaleable CO₂-responsive smart separation membrane (PPFM) prepared by capillary force-driven confined field self-assembly (CFCS) strategy (Fig. 9g). The membrane achieves reversible switching of surface wettability between superhydrophobic/underwater superoleophilic and superhydrophilic/underwater superoleophobic under CO_2_/N_2_ stimulation and has been successfully used for highly efficient separation of a wide range of oil–water systems, including immiscible mixtures, surfactant-stabilized emulsions, multiphase emulsions, and contaminant-containing emulsions (Fig. 9b–f). The membrane exhibits high separation efficiency (> 99.9%), excellent cycling stability, and self-cleaning performance, which provides a more feasible technological path for CO_2_-responsive membranes to move from the laboratory to practical applications [185]. However, the polyester fabric substrate was used, which was slightly less biodegradable. Yuan et al. reported CO_2_-responsive nanofiber membranes (SNMs) prepared by electrostatic spinning technique, which consisted of PMMA-co-PDEAEMA copolymers (Fig. 9j), and were able to achieve reversible switching of surface oil–water wettability between hydrophobicity/lipophilicity and hydrophilicity/lipophobicity under the stimulation of CO₂/N₂ alternation (Fig. 9k). Its nanofiber structure and high roughness significantly enhance the wettability regulation, and the membrane exhibits high oil flux (17,000 L m^−2^ h^−1^) and good separation efficiency (less than 30 ppm oil in water), and excellent mechanical stability and can be reused many times, which provides a new idea for the design of green-gas-triggered smart separation materials. It provides a new idea for the design of green gas-triggered smart separation materials [186]. In summary, gas-responsive materials primarily achieve wettability switching based on the reversible reaction between amine groups and CO_2_, also including a few systems sensitive to gases such as O_2_, SO_2_, and NH_3_ [187]. CO_2_-responsive polymers can be synthesized in batches via free radical polymerization, but the gas response process typically requires a closed system, increasing the complexity of the setup [188]. The polymer chains undergo volume changes during repeated protonation/deprotonation cycles, and cross-linking treatments can effectively enhance mechanical stability, enabling dozens of cycles without significant performance degradation. As a trigger, CO_2_ is widely available, produces no secondary pollution, and can also be utilized from industrial waste gas for resource recovery. Gas-responsive materials are suitable for industrial scenarios with CO_2_ sources, confined spaces, and food processing wastewater treatment. Compared with electro-responsive materials, gas-responsive materials require no wiring and offer simple operation, but their response speed is relatively slower, and they require support from a gas supply system.

### Ion-Responsive Smart Materials

The wettability control technology of smart materials has formed a complete technical system from light/electric response, gas response to ion response. While gas-responsive materials realize wettability control through environmentally friendly triggers such as CO_2_, ion-responsive smart materials provide another precise control pathway for oil–water separation through the interaction of surface charged groups with specific ions. Unlike the gas response that relies on gas molecules for triggering, ion-responsive materials are able to undergo significant and reversible changes with the specific ion species, concentration, or valence state in the environment and reversibly regulate the surface wettability through ionic strength or counter-ion exchange [189]. These materials are able to “sense” and “respond” to the ionic signals in solution and convert chemical information into the switching of surface physical properties, which is one of the key materials to realize the intelligent regulation of solution environment. The core principle is that the functional groups on the surface of the material interact specifically or non-specifically with the ions in solution, triggering changes in molecular chain conformation, charge state, or interfacial energy, which is macroscopically manifested as a dynamic change in contact angle. This regulatory mechanism based on ion–molecule interactions expands the application of smart materials in special environments such as seawater desalination and high-salt wastewater treatment [190].

**Fig. 10:**
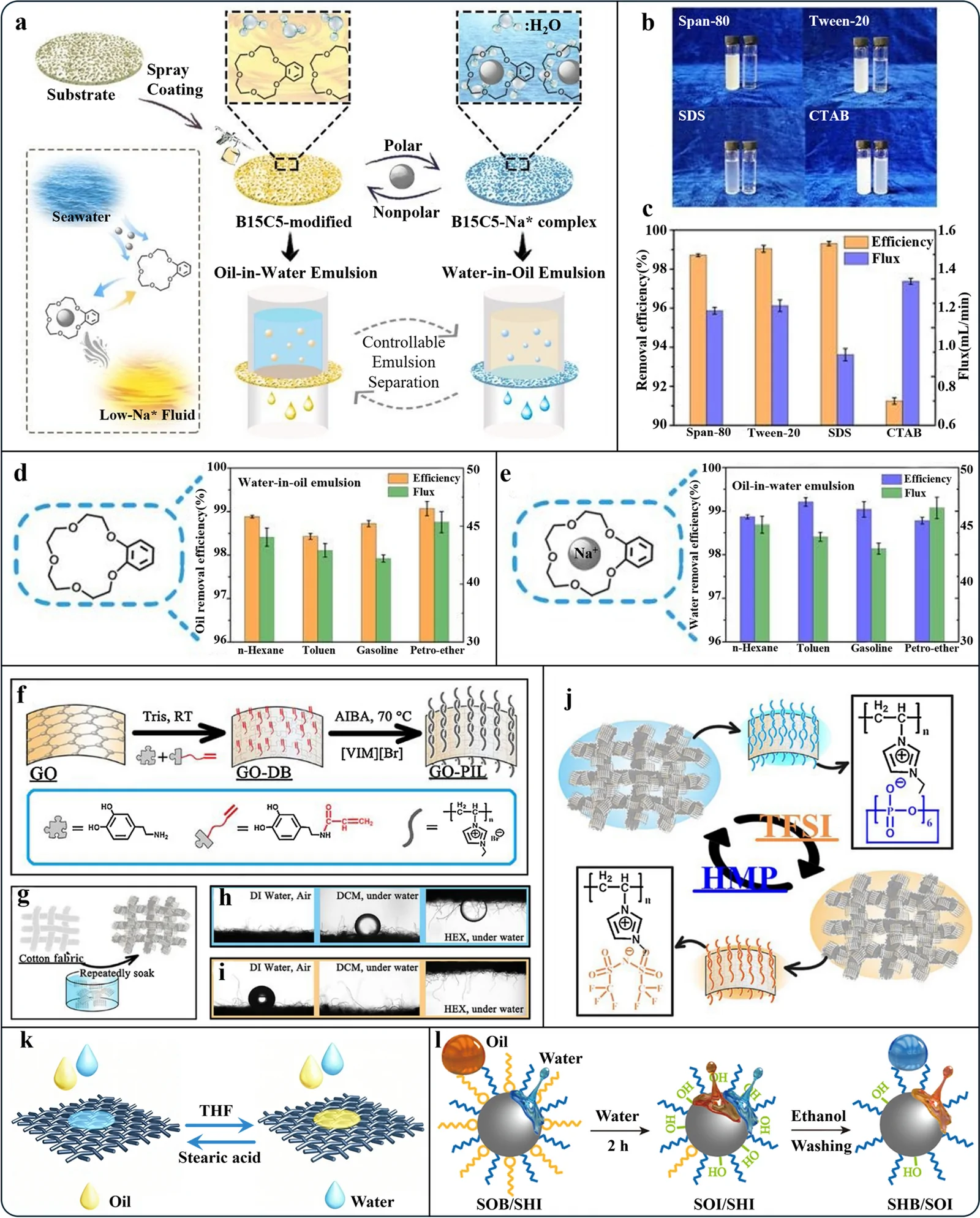
**a** Schematic representation of the separation of Na^+^-responsive controllable emulsions by B15C5-coated membranes. In the absence of Na^+^, B15C5 is nonpolar neutral and has a low surface energy, so the prepared membrane is highly hydrophobic and superlipophilic, realizing the separation of W/O emulsions. When Na^+^ is present, the Na^+^ crown ether coordination complex is polar and positively charged. The membrane transformed into highly hydrophilic/underwater superoleophobic, realizing the separation of various O/W emulsions. **b** Na^+^-controlled emulsion separation studies: digital photographs of various surfactant-stabilized feed emulsions and corresponding filtrates; **c** effect of different surfactants on the separation efficiency and flux of four emulsions; **d** separation efficiency and flux of various W/O emulsions stabilized by Span 80; **e** separation efficiency and flux of various O/W emulsions stabilized by Tween 20. Reproduced from Ref. [191] with permission, copyright 2020, Royal Society of Chemistry. Production schematics for** f** GO-PIL and **g** PIL-CF. Water contact angle and oil contact angle photographs of **h** [GO-PIL@CF] [HMP] and** i** [GO-PIL@CF] [TFSI]; **j** schematic diagram of switchable hydrophilic and hydrophobic properties. Reproduced from Ref. [192] with permission, copyright 2020, Elsevier BV. **k** Schematic diagram of the prepared mesh with reversible wettability switching for controllable oil/water separation. After modification with stearic acid, oil can permeate through the mesh pores, while water cannot. After desorption with tetrahydrofuran (THF), water can rapidly permeate through the mesh pores, while oil cannot. Reproduced from Ref. [193] with permission, copyright 2014, American Chemical Society. **l** Schematic diagram of the wettability transition from superoleophobic/superhydrophilic to superhydrophobic/superoleophilic. Reproduced from Ref. [194] with permission, copyright 2021, American Chemical Society

Oil–water emulsions (including O/W and W/O types) formed in the ocean are highly stable and difficult to handle efficiently, while existing smart superwetting materials (e.g., light-, pH-, and temperature-responsive types) have limited applications in the marine environment due to the lack of effective external stimuli. Feng et al. designed a Na^+^-responsive for oil–water separation in seawater environments by modifying benzo-15-crown-5 (B15C5) on polytetrafluoroethylene (PTFE) membranes via spraying method. The membrane exploits the induced crown ether polarization effect of B15C5 on the cationic electric field to exhibit high hydrophilicity/underwater superoleophobicity in the presence of Na^+^, effectively blocking oil droplets in Na^+^-rich water; after Na^+^ detachment, the membrane recovers its high hydrophobicity/superoleophilicity, blocking water permeation (Fig. 10a). This design provides the membrane with tunability and high adaptability, with the advantages of reusability, high separation efficiency, and sensitive response (Fig. 10b–e) [195]. However, its functional singularity (only responding to Na⁺) and the limitation of being dependent on specific ion concentrations still limit the expansion of its application to some extent. In contrast, Zhao et al. designed a novel ionic species-responsive oil–water separation material: a poly (ionic liquid) (PIL) was loaded onto graphene oxide (GO) via free radical polymerization, and the PIL-modified GO lamellae (GO-PIL) were subsequently coated onto cotton fabrics (CF) (Fig. 10f, g). The wettability of GO-PIL@CF can be improved by anti-anion exchange to switch between hydrophilic and hydrophobicity, enabling efficient separation in oil–water mixtures. In the hydrophilic state, water is permeable while oil is blocked; the opposite is true in the hydrophobic state (Fig. 10h–j) [192]. Due to the loose fiber structure of the material, which provides good permeation flux, the separation process can be driven by gravity without additional energy input, thus reducing energy consumption and simplifying operation. In addition, GO-PIL@CF has soft and durable mechanical properties that make it suitable for use as a conventional absorbent wrap or directly as an absorbent for the selective removal of water or oil from mixtures. In summary, ion-responsive materials are primarily based on specific recognition groups such as crown ethers and poly (ionic liquid)s, achieving wettability modulation through ion exchange or coordination interactions. The synthesis processes for functional molecules like crown ethers are relatively complex, which limits the large-scale preparation of these materials. However, they can be loaded onto porous substrates via methods such as spraying or dip coating to realize functional applications [196]. The wettability transition driven by ion exchange exhibits good reversibility, but the response rate is limited by the ion diffusion process. The preparation process typically avoids organic solvents, making these materials suitable for seawater environments and high-salinity industrial wastewater [197]. Compared with gas-responsive materials, ion-responsive materials can utilize naturally occurring ions in the environment (e.g., Na⁺ in seawater) as triggers. However, their response speed is slower, and achieving selective modulation is more challenging.

### Solvent-Responsive Smart Materials

Facing complex industrial wastewater systems, the response dimension of smart materials has expanded to include solvent environment regulation, and utilizing existing solvents as triggers is also an effective strategy. With the continuous development of stimulus types, solvent-based triggers such as organic solvents, hygro solvents, and ethanol are gradually being exploited for the construction of smart wettability materials [30, 198]. The core working principle of solvent-responsive materials relies on solvent-sensitive units introduced in the molecular design, such as amphiphilic block copolymers or dynamic covalent bonding networks [199]. Under stimulation by different solvents, these groups drive the intelligent switching of the material’s surface wettability by undergoing conformational rearrangements, solubility mutations, or bulk phase transitions.

In the field of oil–water separation, solvent-responsive materials exhibit unique application advantages. For instance, upon stimulation by ethanol or specific organic solvents, some smart membrane materials can achieve a rapid and reversible transition between hydrophilic and hydrophobic properties, thereby switching between “oil-removing” and “water-removing” separation modes on demand [200]. Such materials are particularly suitable for scenarios where a specific solvent environment exists on an industrial site, as the solvents already present in the process stream can be used directly as triggers without introducing additional chemical reagents, offering advantages of simple operation and rapid response [201]. Furthermore, solvent-responsive materials show promising application prospects in areas such as biodiesel purification (e.g., methanol-responsive membranes) and switching the adsorption modes of oil-absorbing materials. Their rapid response and environmental adaptability grant them unique advantages in industrial separation and environmental remediation [202]. Liu et al. designed a copper mesh separation material capable of reversible wettability switching through solvent manipulation [193]. In their experiment, a Cu(OH)_2_ nano-needle array was first constructed on the copper mesh surface via chemical oxidation, forming a superhydrophilic/underwater superoleophobic surface. Subsequently, this copper mesh was immersed in an ethanolic stearic acid solution for 5 min. Through self-assembled monolayer modification, the surface transitioned to a superhydrophobic/superoleophilic state. More importantly, immersing the modified copper mesh in THF for 5 min, leveraging the high solubility of stearic acid in THF, rapidly removed the self-assembled layer, restoring the surface to its superhydrophilic/underwater superoleophobic state. This process was cyclable multiple times with almost no change in surface morphology. Based on this solvent-regulated reversible wettability switching, the copper mesh achieved controllable separation of oil–water mixtures. In the superhydrophobic state, the oil phase could rapidly permeate while water was blocked. After THF treatment and transition to the superhydrophilic state, the water phase could permeate while oil was blocked (Fig. 10k). This provides a simple, rapid, and repeatable solvent-regulating strategy for intelligent oil–water separation materials. Although the solvent-responsive copper mesh developed by Liu et al. achieved simple and rapid reversible wettability switching through stearic acid self-assembly and THF desorption, providing an efficient regulation strategy for intelligent oil–water separation, its functionality primarily relies on the physical adsorption and desorption of a single small molecular layer. This poses a risk of molecular layer depletion during long-term cyclic use, and its separation capability for complex emulsion systems is limited. In contrast, the TiO_2_/perfluorooctanoic acid (PFOA) composite coating constructed by Li et al. innovatively introduces a multi-stage wettability transition mechanism based on solvent responsiveness [194]. The wettability switching mechanism is based on the regulation of surface polar components: initially, the low-surface-energy fluorocarbon chains provided by PFOA impart oleophobicity, while the NH₄⁺ hydrophilic groups provide hydrophilicity. Upon immersion in water, the NH₄⁺ is lost, and –OH groups formed on the TiO_2_ surface transition the coating to a superoleophilic/superhydrophilic (SOI/SHI) state. After ethanol washing removes the –OH groups, the residual PFOA renders the surface superhydrophobic/superoleophilic (SHB/SOI) (Fig. 10l). Based on this, the superoleophobic/superhydrophilic (SOB/SHI) state can separate light oil–water mixtures and oil-in-water emulsions, while the SHB/SOI state can separate heavy oil–water mixtures and water-in-oil emulsions, both achieving separation efficiencies > 99.9%. Additionally, the TiO₂ in the coating can catalyze the decomposition of PFOA under UV light, reducing fluorochemical pollution. This research provides a new approach for developing environmentally friendly, functionally tunable superwetting materials.

In summary, the performance of solvent-responsive materials is based on solvent-induced conformational transitions or surface molecular rearrangement. Self-assembled monolayer modification methods are simple and rapid, but the molecular layer may gradually desorb during long-term cycling. Dynamic covalent networks or polymer brush structures offer greater stability, but their preparation is more complex [203]. Solvent-induced conformational transitions exhibit good reversibility; however, long-term cycling may lead to gradual performance degradation due to solvent residue or depletion of the molecular layer. The use of organic solvents increases process complexity, but these materials can leverage existing solvents in the process stream as triggers. This makes them suitable for industrial sites with specific solvent environments, such as in the chemical and pharmaceutical industries.

### Multi-Responsive Smart Materials

In increasingly complex oil–water mixing environments, smart oil–water separation materials that respond only to a single stimulus often struggle to cope with the complex conditions and may even fail. Therefore, multi-responsive smart materials with tunable wettability have been widely investigated, such as combinations of heat/pH, heat/light, pH/light [204], CO_2_/N_2_, ion/heat, magnetic/electric field, pH/NH_3_, and heat/pH/glucose concentration. Multi-responsive smart materials can change their affinity for water or oil by modulating the surface chemical properties or the surface micro-nanostructure [26, 205].

**Fig. 11:**
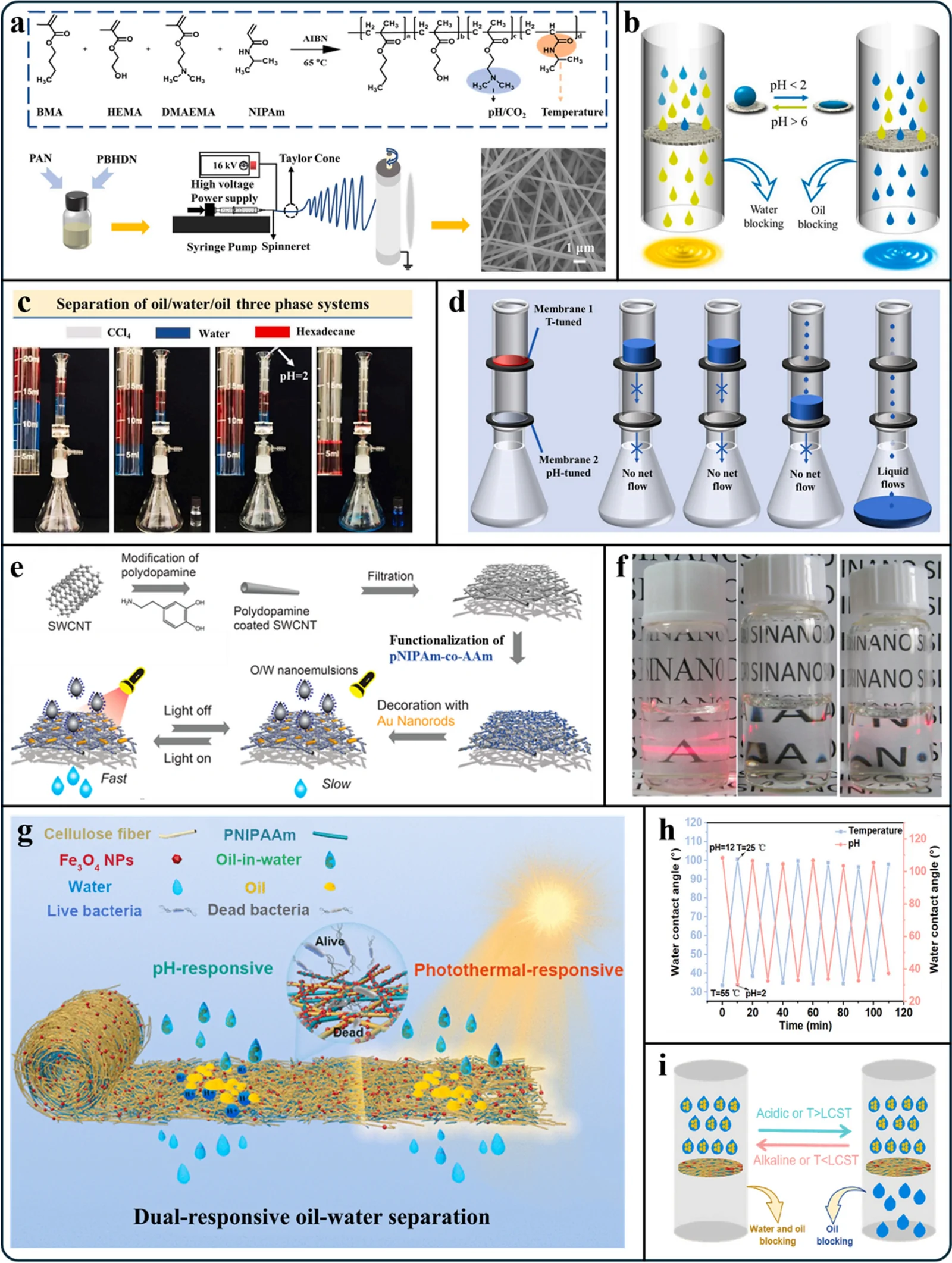
**a** Synthesis and preparation of PBHDN /PAN membranes. Oil–water separation performance of membrane. (**b**: Schematic diagram of pH-responsive separation **c**: photograph showing the continuous oil–water-oil three-phase separation.) **d** Device schematic of two-layer membrane utilizing PBHDN/PAN membrane to realize logic and gate regulated by temperature and pH. Reproduced from Ref. [206] with permission, copyright 2022, Elsevier BV. **e** Schematic diagram of the preparation of photothermally responsive gold nanorods/pNIPAm-co-AAm co-hybrid SWCNTS ultrathin films. **f** Corresponding photographs of the original H/W, the filtrate separated in the light off and on. Reproduced from Ref. [207] with permission, copyright 2015, American Chemical Society. **g** Schematic diagram of ECF-P-Fe_3_O_4_-N membrane. **h** Reversible cycle stability of water wettability of ECF-P-Fe_3_O_4_-N in air. **i** Schematic diagram of pH/temperature-responsive separation. Reproduced from Ref. [208] with permission, copyright 2025, Elsevier B.V

Temperature/pH-responsive materials can selectively adsorb or repel water or oil by realizing a transition from hydrophilic to hydrophobic surfaces at different temperatures or pH conditions. Dou et al. synthesized reactive copolymers using isopropylacrylamide and 2-(dimethylamino) ethyl methacrylate and prepared smart composite membranes with polyacrylonitrile (PAN) by electrostatic spinning (Fig. 11a). The membranes had tunable wettability and were able to switch between hydrophobicity and hydrophilicity by temperature and carbon dioxide to achieve on-demand separation of oil–water/oil three-phase mixtures. In the initial state, the membrane is hydrophobic to oil and effectively blocks water, whereas, in the presence of acidic water or carbon dioxide, the tertiary amine groups in the copolymer are protonated, and the membrane surface properties change to promote water permeation and achieve reverse separation (Fig. 11b, c). In addition, the structure of the membrane is reversibly adjusted with temperature, and water is able to selectively pass through the membrane after heat treatment. By virtue of the temperature and CO_2_ response properties, the membrane can efficiently separate different types of oil–water mixtures and emulsions (Fig. 11d) [206]. Its excellent separation effect and controllability make it a potential for a wide range of applications in the fields of oil–water separation, oil wastewater treatment, and energy and environmental protection. However, this membrane relies on pH or temperature changes for regulation, which may be limited in scenarios requiring rapid, remote control, and its flux (up to 241.9 L m^−2^ h^−1^) is relatively low compared to membranes designed for high-speed separation. Hu et al. proposed an innovative method to construct an ultrathin single-walled carbon nanotube (SWCNT) network membrane co-hybridized with gold nanorods (ANR) and poly(n-isopropylacrylamide-acrylamide) (pNIPAm-co-AAm), which was applied to the ultrafast separation of O/W nanoemulsions. The membrane was able to precisely regulate the permeation flux by light for on/off switching separation. The membrane construction process consisted of four steps: first, SWCNT was modified with dopamine; next, the membrane surface was decorated using a polydopamine coating; then, pNIPAm-co-AAm copolymer was introduced to enhance the thermal response properties; and finally, gold nanorods (ANRs) were added to improve the photothermal effect (Fig. 11e). The membrane possesses excellent hydrophilicity, oleophobicity, and tunable nanopore size, which enables efficient separation of nano-emulsion with a flux of up to 35,890 m^2^ h^−1^ bar^−1^ (Fig. 11f) [207]. During the separation process, light can precisely regulate the permeation flux of the membrane, and the separation efficiency of the membrane is more than 99.99%, with good anti-fouling performance and recyclability, showing great potential for application in nanoemulsion separation. However, this membrane relies on light stimulation, which may be limited in dark environments, and its fabrication involves relatively complex nanomaterial modification steps. In contrast, Xu et al. successfully prepared an electrospun cellulose nanofiber membrane (ECF-P- Fe_3_O_4_-N) with photo-thermal/pH-responsive wettability, which integrates three interpenetrating network formation of cellulose and thermal polymer, co-precipitation embedding of photo-thermal mediators (Fe_3_O_4_ nanoparticles), and chemical grafting of aminosilane key strategies (Fig. 11g). The membranes exhibit high hydrophilicity under acidic or high-temperature stimulation and hydrophobicity under alkaline or low-temperature stimulation and can rapidly and reversibly switch wettability to separate a wide range of immiscible oil–water mixtures and surfactant-stabilized emulsions to meet different needs (Fig. 11h, i) [208]. The maximum separation flux for O/W emulsions is up to 225.6 L m^−2^ h^−1^ with a separation efficiency of over 98.53%. At the same time, the membrane also has a strong sterilization ability to meet the needs of long-term contact use, which is of great significance in the field of remote control oil–water separation.

Smart response materials show excellent performance in oil–water separation, but the environmental impact of their full life cycle cannot be ignored. Zhuang et al. pointed out that traditional polymers are difficult to degrade and are prone to secondary pollution such as microplastics after disposal [209]. However, materials based on natural polymers such as chitosan (CS) have good biocompatibility and degradability, and their degradation products are mostly non-toxic small molecules, which can participate in the natural carbon cycle and reduce the environmental load. However, there is still a lack of systematic research on the long-term degradation behavior, ecotoxicity of degradation products, and environmental fate of these materials, and Ejeromedoghene et al. further emphasized that the environmental impacts of hydrogel materials run through the whole life cycle of raw materials-preparation-use-disposal, which may involve fluorine compounds or organic solvents [210]. The preparation process may involve fluorine-containing compounds or organic solvents, the use process can reduce the consumption of chemical cleaning agents through anti-pollution design, and the disposal process needs to take into account the possible microplastic contamination caused by non-biodegradable substrates. Therefore, the environmental impact assessment of smart response materials should start from the perspective of the whole life cycle and take into account the renewability of raw materials, the greening of the preparation process, the prolongation of the use cycle, and the controllable degradation at the disposal stage while pursuing the efficient separation performance, so as to realize the unity of environmental remediation and eco-friendliness. On the basis of the above environmental considerations, despite the remarkable progress in the research of smart response wetting materials, there are still many challenges that need to be addressed. For instance, industrial applications are limited by the lack of long-term stability, the high cost of practical applications, and the complexity of large-scale preparation processes. Future research should be focused on the development of low-cost, highly durable multi-responsive materials and the optimization of stimulation response efficiency. Meanwhile, new directions for achieving smart and sustainable oil–water separation are provided by simplifying the preparation process and promoting the integration of materials with AI technologies. Based on the above research, the preparation of multi-responsive materials typically requires multi-step modification or composite processes, making the procedure complex [211]. However, scalability can be improved through modular design. The synergistic mechanism of multiple stimuli enables more stable responsive behavior: failure of a single stimulus can be compensated by others, resulting in overall chemical stability superior to single-response systems. Composite material design can balance the mechanical properties of different components, but interfacial bonding strength is a key factor affecting long-term stability. These materials can be designed to be fully bio-based, with preparation processes avoiding toxic solvents [212]. They are suitable for complex industrial wastewater, environments with multiple parameter variations, and intelligent sensing systems. Compared with single-response mechanism materials, multi-responsive materials adapt to more complex environmental conditions and offer higher regulation precision [213]. However, their preparation is more complex and costly, requiring coordination among multiple stimulus sources for synergistic operation.

### Stimulus-Responsive Catalytic Cleaning Membranes

The core principle of stimulus-responsive catalytic cleaning membranes for oil–water separation lies in the reversible regulation of surface wettability and in situ degradation of pollutants through the integration of smart polymers with environmentally responsive properties (e.g., temperature and light, CO_2_, etc.) and catalytically active nanomaterials (e.g., Ag/AgCl, TiO_2_, and MXene, etc.) in a porous substrate [214–216]. During the separation process, the membrane material responds to external stimuli and undergoes changes in molecular conformation or surface chemistry, thus intelligently switching between “superhydrophobic/superoleophilic” and “superhydrophilic/superoleophobic underwater” to realize selective separation of the oil or water phase. At the same time, the loaded catalyst generates active oxygen species under the stimulation of light or other stimuli, which completely mineralizes the oil and organic dyes adsorbed or clogged on the surface of the membrane and in the pores, giving the membrane long-lasting self-cleaning performance [217]. This technology shows a broad application prospect in the fields of complex oily wastewater treatment, emulsion separation, and dye removal. In specific applications, different types of stimuli-responsive membranes are distinctive and complementary to each other. For example, the Fe/TiO_2_ superhydrophobic membrane prepared by Gao et al. through dynamic etching extended the photoresponsive range of TiO_2_ to the visible light region by using Fe doping and could realize simultaneous oil–water separation and photocatalytic degradation of dyes under light, with an average separation efficiency of 96.4%, and the hydrophobicity was enhanced and the separation efficiency was increased to 96.81% after light illumination; however, it relied on a single light-responsive mechanism, and its application was limited under the no-light condition. However, it relies on a single light-responsive mechanism, which limits its application in the absence of light [167]. In contrast, the PNIPAM@PVDF-Fe/contact angle temperature-sensitive membrane developed by Wu et al. utilized the bulk phase transition to regulate the membrane pore size and wettability by grafting PNIPAM hydrogel on the surface, and the flux recovery rate reached 97.29% under the cyclic cleaning of 30–50 °C, which showed excellent temperature-controlled self-cleaning performance, but high temperature was easy to lead to the expansion of the membrane pore, which reduced the pollutant removal efficiency. However, high temperature is easy to cause membrane pore expansion, reducing the pollutant removal efficiency [145]. It can be seen that the light-responsive membrane has excellent dye degradation ability under visible light drive, while the temperature-sensitive membrane has outstanding performance in temperature-controlled self-cleaning, and both of them can be selectively applied according to the light and temperature conditions of the actual scene.

In the design of stimulus-responsive catalytic cleaning membranes, the hydration phenomenon and the catalytic mechanism do not exist independently of each other, but are synergistically coupled through interfacial chemical and physical processes, which together determine the separation efficiency, contamination resistance, and regeneration performance of the membrane. Hydration refers to the process of water molecules forming an ordered hydration layer on a hydrophilic surface through hydrogen bonding, electrostatic interactions, or ligand bonding. This hydrated layer can act as a physical barrier to prevent oil droplets or organic pollutants from directly contacting the membrane surface, thus significantly reducing the adsorption and adhesion of pollutants. However, when contamination of the membrane surface inevitably occurs, catalytic mechanisms provide a way to actively remove the contaminants [171, 218, 219]. For example, metal active sites (e.g., Mn^3^⁺, Fe^2^⁺/Fe^3^⁺, and Mo^6^⁺, etc.) in the mineral or hydrogel layer can be stimulated by external stimuli (e.g., H_2_O_2_ [220], PMS, light) to generate reactive oxygen species or bubbles [221], which can realize the degradation of contaminants or physical stripping. There are four levels of synergistic coupling between the hydration layer and the catalytic mechanism: first, the stable hydration layer can act as a molecular buffer layer to protect the catalytic active sites from direct coverage by pollutants, thus maintaining the long-term stability of the catalyst (e.g., MnO_2_ membranes maintain high catalytic activity after prolonged separation) [222]; second, the catalytic reaction removes the adsorbed organic matter on the surface of the membranes and restores the surface hydrophilicity, which enable the hydration layer to be reconstructed (e.g., the water contact angle of MnO_2_ membrane is restored to 0° after H_2_O_2_ cleaning) [223, 224]; third, the catalytically generated bubbles disturb the interface during the ascent process, accelerating the rearrangement of water molecules to form a new hydration layer, so that the characteristic recovery time of catalytic cleaning (0.93 min) is much shorter than that of hydrodynamic cleaning (4.14 min) [225]; and lastly, high polarization and oxygen vacancies on the membrane surface enhance the hydration capacity while providing more active sites for the catalytic reaction, which enhances the generation efficiency of reactive oxygen species (e.g., Mn^3^⁺ content is positively correlated with the rate of –OH generation, and Mo^6^⁺ modulation of Fe^2^⁺/Fe^3^⁺ cycling enhances peroxynitrite activation) [226]. The intrinsic connection between hydration phenomenon and catalytic mechanism constitutes the core scientific foundation of stimulus-responsive catalytic cleaning membranes: the hydration layer forms a passive antifouling barrier, and the catalytic reaction gives active regeneration ability, and the two are synergistically coupled through the interface to realize the integrated function of “separation + self-cleaning”. From the perspective of wettability theory, the formation of a hydrated layer is essentially a transition of the surface from the Wenzel [38] or Cassie–Baxter [41] state to a special “hydrophilic Cassie state”. Water molecules are locked in the rough structure to form a molecular barrier, with a very low contact angle hysteresis (*Δθ* = 0°) [45], making it difficult for contaminants, such as oil droplets, to adhere, and giving the material excellent resistance to contamination. When contamination occurs, the catalytic mechanism is activated and reactive oxygen species degrade the contaminants, rebuilding the interfacial structure of the Cassie–Baxter state at the molecular level and restoring the contact angle to nearly 0°. The characteristic recovery time of catalytic cleaning (0.93 min) was much shorter than that of hydrodynamic cleaning (4.14 min), which was attributed to the fact that the catalytic reaction proactively rebuilt the interfacial structure of the Cassie–Baxter state from the molecular level and enhanced the separation stability. This significantly improves the separation efficiency, pollution resistance, and durability of membrane materials in complex water bodies. Future research should focus on exploring the influence of light-heat-electricity-chemistry and other multi-field coupling response mechanisms on hydration-catalytic synergy.

To systematically summarize the above discussion, Table 1 organizes the key performance parameters (type of materials, stimulus type, wettability, separation efficiency, and flux) of representative materials in order to visually compare the performance differences of different material systems. On this basis, Table 2 compares the core features of various types of stimulus response mechanisms in terms of response speed, regulation precision, reversibility, energy input, and energy consumption which provides a reference for researchers to select the appropriate response mechanisms according to the actual application requirements.

**Table 1:** Comparison of various material properties

Type of material	Stimulus type	Wettability	Separation efficiency (%)	Flux (L m^−2^ h^−1^)	Refs
Nylon membrane	Tempe	WCA: 40°/UOCA: 153° (25 °C)	99% at 25 °C, O/W emulsion	> 1500, O/W emulsion	[143]
		WCA: 120°/OCA: 0° (45 °C)	99% at 45 °C, W/O emulsion	> 900, W/O emulsion	
Tung oil-derived spongy	PH	WCA: 142.5°/UOCA: 0° (pH = 7)	99.9%, O/W emulsion	6,700, O/W emulsion	[154]
		WCA: 0°/UOCA: 137.1° (pH = 13)			
Photocatalytic fibrous membrane	PH	WCA: 130° (pH ≤ 7)	99.2%, oil–water mixture	10,000, oil–water mixture	[157]
		WCA: 0°/UOCA: 145° (pH = 11)			
ZnO hybrid membranes	Light	WCA:11.09°/UOCA: > 150°	> 99%, emulsion	338.2, emulsion	[167]
Fe/TiO_2_ composite membrane	Light	WCA: 140.16° (before illumination)	98.17%, oil–water mixture	27,346.27, oil–water mixture	[168]
		WCA: 153.37° (after illumination)			
TENG	Electric	–	96.97%, W/O emulsion	–	[175]
Stainless-steel mesh	Electric	WCA: 159°/UOCA: 155° (initial)	99.14%, oil–water mixture	360, oil–water mixture	[177]
		WCA: 40°/UOCA: 155° (energized)			
Nanofibrous membranes	Gas	WCA: 142.1°/OCA: 0° (initial), WCA: 0°/UOCA: 145° (CO₂-stimulated)	99.99%, oil–water mixture	12,865, oil–water mixture	[186]
		WCA: 142°/OCA: 0° (N_2_-treated)	99.83%, emulsion	5015, emulsion	
Polyester fabric membrane	Gas	WCA: 140°/UOCA: 0° (initial)	99.9%, oil–water mixture	325, O/W emulsion	[187]
		WCA: 0°/UOCA: 150° (CO_2_)	99.5%, emulsion		
		WCA: 140° (N_2_)			
Crown ether Na⁺-responsive membrane	Ion	WCA: 145°/OCA: 0° (initial)	99.08%, W/O emulsion	45.3, W/O emulsion,	[193]
		WCA: 20°/UOCA: > 150° (with Na⁺)	99.21%, O/W emulsion	44.0, O/W emulsion	
GO-PIL@CF	Ion	WCA: 0°/UOCA: 147° (HMP)	> 98%, oil–water mixture	59,000, O/W emulsion	[194]
		WCA: 148°/UOCA: 0° (TFSI)			
Modified copper mesh	solvent	WCA: 0°/UOCA: 158.5° (oxidized)	–	–	[195]
		WCA: 155.4°/OCA: 0° (ethanolic stearic acid)			
		WCA: 0°/UOCA: 158.5° (THF)			
TiO_2_/PFOA hybrid coating	solvent	WCA: 0°/UOCA: 150° (SOB/SHI)	99.9%, oil–water mixture	9,172, oil–water mixture	[206]
		WCA: 178.9°/UOCA: 0° (SHB/SOI)	99.9%, emulsion	70 O/W emulsion	
				62, W/O emulsion	
Electrospun membrane	PH/Tempe/Gas	WCA: 0° (acidic), WCA: 130° (alkaline), WCA: 130° (25°C),	95–99.6%, Emulsion	241.9, emulsion	[211]
		WCA: 128° (50°C)			
		WCA: 61.32° (pH = 2)			
Cellulose nano fiber membrane		WCA: 0°/UOCA: 145° (pH = 10)	98.53%, O/W Emulsion	225.56, O/W Emulsion	[213]
	PH/Tempe	WCA: 100° (25°C)			
		WCA: 30° (55°C)98.53%, O/W emulsion, 225.6, O/W emulsion

**Table 2:** Comparison of various response material indicators

Responsive mechanisms	Response speed	Regulation precision	Reversibility	Energy input	Energy consumption	Refs
Thermo-responsive	Fast (seconds)	Precise control via temperature gradient	Excellent	Thermal energy (heating/cooling)	Medium–high	[142–144]
PH-responsive	Fast (seconds)	Precise regulation by pH value	Excellent	Chemical energy (acid/base solution)	Low	[152–155]
Photo-responsive	Medium (minutes)	Control through light intensity/illumination time	Good	Light energy (UV/Vis)	Low	[165–168]
Electric-responsive	Fast (seconds)	Precise regulation by voltage/current	Excellent	Electrical energy	Medium–low	[173–176]
Gas-responsive	Medium (minutes)	Control via gas type/time	Excellent	Gas (CO₂/N₂)	Low	[183–186]
Ion-responsive	Slow (hours)	Regulation by ion type/concentration	Good	Chemical energy (salt solution)	Low	[191, 192]
Solvent-responsive	Fast (seconds)	Control through solvent polarity/type	Good	Chemical energy (organic solvent)	Medium–low	[193, 194, 199, 201]
Multi-responsive	Adjustable (seconds to minutes)	Multi-parameter synergistic regulation	Excellent	Combination of multiple energy types	Medium–high	[206–208]

The criteria for evaluating reversibility are as follows: excellent means the material maintains excellent performance stability after undergoing multiple stimulus response cycles. Good means the material exhibits good reversibility, but with some performance degradation under long-term or extreme conditions. The criteria for evaluating energy consumption are as follows: low: requires only an instantaneous trigger (e.g., light exposure, a small amount of solution, gas introduction) without the need for continuous energy input to maintain the responsive state; the trigger source itself has low energy consumption (e.g., visible light, atmospheric pressure gas); or it can utilize ambient/waste energy (e.g., industrial waste gas CO_2_). Medium–low: requires instantaneous energy input, but the trigger source has slightly higher energy consumption (e.g., low-voltage electric field, organic solvent); or the response process has low energy consumption but the energy consumption of auxiliary processes needs consideration (e.g., solvent recovery, solution replacement). Medium–high: requires continuous energy input to maintain the responsive state (e.g., heating/cooling); or requires relatively high energy for triggering (e.g., high-voltage electric field, UV light source). High: requires continuous high-energy input; or the synergistic effect of multiple energy sources leads to a significant increase in overall energy consumption

## Smart Response to the Preparation of Wetting Materials

In recent years, with the development of smart materials and nanotechnology, methods for the preparation of smart-responsive surfaces have become progressively more diverse and efficient. These methods are able to precisely regulate surface roughness, chemical composition, and wettability, enabling materials to respond under specific environmental conditions [227]. Compared to inorganic materials, functionally responsive polymers are preferred for the preparation of smart surfaces due to their ability to precisely control surface energy and morphology at the molecular level. Functional responsive polymers are able to respond rapidly to external stimuli (e.g., temperature, light, pH, solvent, gas, etc.) and have good reversibility [228]. In the design of wettability-responsive smart surfaces, various stimulus-responsive units can be embedded to precisely regulate the surface properties [229]. These smart surfaces are not only flexible and adaptable, but also show great potential in various fields, such as sensors, smart coatings, smart textiles, drug delivery systems, and water treatment. Here, we only introduce the main methods that have been recently used to prepare polymer-based smart-responsive surfaces, such as layer-by-layer self-assembly, electrospinning, and surface-initiated atom transfer radical polymerization (SI-ATRP).

### Layer-by-Layer Self-Assembly Methods

Self-assembly is a process of spontaneous formation of ordered aggregates based on non-covalent interactions between molecules (e.g., electrostatic attraction, hydrogen bonding, and van der Waals forces, etc.) [63]. This method has significant advantages such as high efficiency, low cost, and tunability. It is capable of constructing smart surfaces with specific functions without relying on complex multi-step processes and has strong self-healing ability and structural stability [230]. Layer-by-layer (LBL) self-assembly technology, developed on this basis, is a precisely regulated method of constructing multilayer films step by step by alternately depositing materials with opposite charges or complementary interactions (e.g., polyelectrolytes and nanoparticles, etc.) on the surface of the substrate. LBL self-assembly inherits the intermolecular interaction mechanism of self-assembly, but further realizes LBL precise control of coating thickness, surface roughness, porosity, and chemical composition. Therefore, it is able to regulate the functional properties of materials such as hydrophilicity, electrical conductivity, and antimicrobial properties more flexibly [231, 232]. The main advantages of this technology are the gentle film formation process, the wide range of applicable substrates, and the strong functional integration. In the field of oil–water separation, the LBL self-assembly technology is widely used to construct superhydrophilic/underwater superoleophobic or superhydrophobic/superoleophilic coatings to achieve efficient separation and degradation of pollutants, such as emulsified oil, dyes, and antibiotics [233–236]. Meanwhile, this technology also provides an ideal platform for the preparation of a variety of smart-responsive materials such as temperature-responsive, pH-responsive, and gas-responsive materials. By assembling responsive polymers or nanomaterials layer by layer onto porous substrates, the surface wettability can be reversibly regulated, thus switching between “oil-removing” and “water-removing” separation modes on demand. Therefore, LBL self-assembly can be regarded as an extension and application of the self-assembly principle in the construction of multilayer structures, which together constitute an important technological basis for the realization of smart-responsive wettable materials from molecular design to macroscopic function [237, 238].

**Fig. 12:**
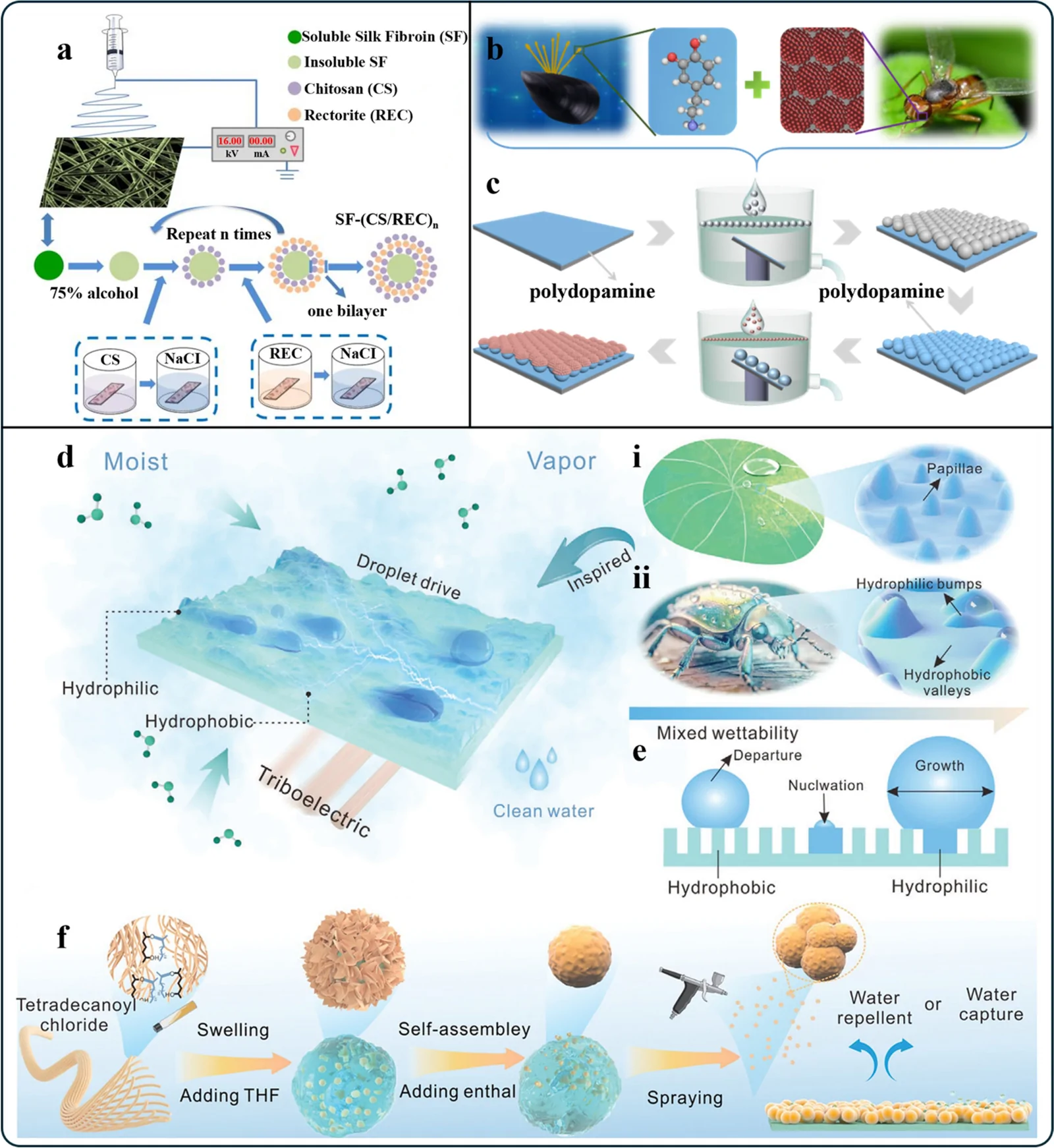
**a** Preparation process of LBL-structured nanofiber mats. Reproduced from Ref. [239] with permission, copyright 2018, Elsevier BV. **b** Mussel mucin is highly adhesive. The ability of mosquitoes to fly in rainy weather depends on the hydrophobic properties of their compound eyes. **c** Preparation process of a layered structured film with superhydrophobic properties. Reproduced from Ref. [240] with permission, copyright 2020, Elsevier BV. **d** Biomimetic superhydrophobic triboelectric surfaces inspired by desert beetles and lotus leaves. **e** Droplet morphology in different regions of a water-collecting surface. **f** Self-assembly design scheme for superhydrophobic surfaces. Reproduced from Ref. [241] with permission, copyright 2018, Wiley

Based on these excellent functional properties, the researchers further explored the potential of the LBL self-assembly technique in constructing multifunctional nanofiber composite membranes for more complex application requirements. Deng et al. modified CS and regolith (REC) onto sericin protein (SF) nano-fibers by LBL self-assembly technology (Fig. 12a) [239] to enhance their physical and biological properties. It was found that the LBL process successfully formed a positively and negatively charged layer on a sericin protein substrate CS is positively charged, REC negatively charged on a composite nanofiber membrane. By adjusting the number of layers and the composition of the outer layers, the morphology, mechanical properties, and their antimicrobial effects of the nanofiber membranes can be adjusted, so that the prepared composite membranes have good wettability and biocompatibility. However, inspired by the compound eyes of mosquitoes (micro-nano-graded structure) and mussels (strong adhesion), Zhao et al. proposed a new method to construct graded-structured superhydrophobic thin films by self-assembly of colloidal particles at the gas–liquid interface. The method first self-assembles single-layer micron-sized polystyrene microspheres on a dopamine-modified substrate and then deposits nanoscale silica particles to form a micro- and nano-graded film with a bright structural color; after treatment with fluorosilane vapor deposition, the film exhibits excellent superhydrophobicity (high contact and low sliding angles) and anti-adhesion, and the change in the structural color intuitively reflects the change in the surface wettability (Fig. 12b, c) [240] and has good performance on both flexible substrates and the films have good applicability on flexible substrates and surfaces with complex shapes, showing potential applications in the fields of sensing and medical catheters. The function of this film is still limited to the field of passive protection. In contrast, Nie et al. inspired by desert beetles (hydrophilic/hydrophobic patterns) and lotus leaves (micro-nano-graded structures) (Fig. 12d), prepared a superhydrophobic friction electric surface with nanoscale hydrophilic domains by interfacial self-assembly for efficient water collection (Fig. 12f). This surface utilizes the self-assembly of amphiphilic cellulose nanoparticles to form micro-/nano-hierarchical structures, enabling rapid droplet nucleation and transport. By integrating the triboelectric effect (which generates electrostatic forces through mechanical inputs such as human walking), the surface actively drives droplet migration and shedding, achieving a 39.02% enhancement in water collection efficiency (Fig. 12e). Furthermore, a portable swing fog collector was successfully fabricated, offering a novel strategy for wearable water harvesting devices in outdoor emergency scenarios [241]. In summary, the main advantages of the LBL self-assembly technique lie in its mild film-forming conditions, wide applicability to various substrates, and the ability to precisely control film thickness, surface roughness, and chemical composition by adjusting the number of layers, components, and assembly sequence, thereby achieving a high degree of functional integration [242, 243]. This method is most suitable for preparing multilayer composite films that require precise control over surface chemical properties, such as pH-responsive materials, gas-responsive materials, and superwetting materials with antifouling functions [244]. However, its limitations include the relatively time-consuming assembly process, the need for further improvement in the long-term stability of the multilayer structures, and certain challenges for large-scale continuous production.

### Electro-Spinning Method

Self-assembly technology provides an important avenue for smart materials development by virtue of its simplicity and high efficiency, while finer structural modulation and functional optimization are often required in combination with other advanced preparation techniques. In this regard, electrostatic spinning technology shows unique advantages [245]. Electrostatic spinning is an advanced technology used to prepare continuous polymer fibers with diameters ranging from the nanometer to the micrometer scale by stretching polymer solutions or melts to form elongated fibers through a high-voltage electrostatic field [246]. This technique allows precise control of the fiber diameter and generates a homogeneous fiber network. Nanofibers prepared by electrostatic spinning have a high specific surface area and adjustable surface roughness, which enhances the hydrophilicity or hydrophobicity of the material and improves reactivity. By adjusting the spinning parameters, the structure and properties of the membrane can also be optimized. These advantages allow electrostatic spinning to show great flexibility in the field of smart response surfaces, where surface properties can be adjusted according to environmental changes (e.g., temperature, humidity, and pH, etc.) [247, 248]. Therefore, electrostatic spinning has become one of the efficient and economical techniques to prepare smart-responsive surfaces, which are widely applied in the fields of sensors, separation membranes, drug release, etc. Zhao et al. designed a superhydrophobic porous SiO₂ micro- and nanofiber membrane prepared by electrostatic spinning combined with freeze-drying technique [249]. The membrane was modified with camphor as the pore-forming agent and polystyrene (PS) as the auxiliary pore-forming polymer by calcination and hexamethyldisilazane (HMDS), and the surface had an elliptical porous structure with a specific surface area about five times that of the conventional SiO₂-fiber membranes, with a water contact angle of more than 150° and a sliding angle as low as 2.1°. With high adsorption capacity (up to 43.7 g g^−1^) and good reusability for a wide range of oils, the membranes show potential for application in oil–water separation and oil spill adsorption treatment. However, this inorganic material-dominated membrane has limitations in terms of biodegradability and environmental friendliness. A biomimetic bilayer-scale biopolymer nanofiber membrane inspired by fish skin was developed by Kwak et al. [250]. A superhydrophilic/underwater superoleophobic separation membrane with a “bead-like” micro-nanocomposite structure was constructed by electrostatic spinning and electrospraying with fish gelatin (FG) as the raw material, and an environmentally friendly ribose cross-linking method was used to enhance its water stability. The membrane shows high efficiency in separating both floating oil and emulsified oil at low pressure of 10 kPa (flux of 2086 L m^−2^ h^−1^, efficiency > 99%) and has excellent reusability and biodegradability in the whole process, which provides a green solution for sustainable oil–water separation. However, its application scenarios are mainly limited to the physical sieving process, which is difficult to cope with the flux attenuation caused by the accumulation of oil on the membrane surface, and it also lacks the active removal capability of dissolved organic pollutants in wastewater. In contrast, the PLA@ZIF-8 composite membrane constructed by Han et al. innovatively introduced the photocatalytic function of ZIF-8 on a biodegradable PLA substrate, which not only achieved higher oil–water separation flux (6280 L m^−2^ h^−1^) and efficiency (> 99.8%), but also could be utilized under UV light. The active oxygen species generated by ZIF-8 effectively degrade organic pollutants such as diesel fuel and methylene blue, solving the problem of membrane contamination from the source [251]. This combination of photocatalytic self-cleaning function and biodegradability enables the membrane material to actively decompose pollutants during use to prolong its service life and can be completely degraded after use to avoid secondary pollution.

Although the electrostatic spinning technique is capable of accurately constructing structurally complex nanofibrous membranes at the laboratory level, the scalability of its industrial production is still the key toward practical applications [252, 253]. Recent studies have demonstrated the scale-up potential through different strategies and exposed their respective considerations. Ma et al. prepared dual-responsive membranes by one-step co-mingled electrospinning introducing PNIPAm and poly(2-(dimethylamino)ethyl methacrylate) (PDMAEMA) with PAN as the substrate, with the advantages of a simple process, no need for post-modification, and ease of scale-up [254]. However, the functional materials are limited to polymers with good compatibility, and the introduction of responsive components affects fiber morphology and mechanical properties, which requires precise regulation of the rheological behavior of the spinning solution in scale-up. Xu et al. used natural cellulose as the raw material and produced a photo-thermal/pH dual-responsive membrane by multistep modification (Interpenetrating Polymer Network (IPN) construction, Fe_3_O_4_ co-precipitation, and aminosilane grafting), which is characterized by the renewable and environmentally friendly nature of the raw material [208]. The highlight is that the raw materials are renewable and environmentally friendly. However, the process is complicated, involves multi-step wet chemical reaction, and faces the challenges of process convergence and cost control in continuous production, which is more suitable for high value-added scenarios. Yang et al. chose industrially matured PVDF and constructed photothermal-responsive membranes through the addition of MXene nano-sheets, which has the advantages of a simple process, compatibility with the existing production line, low amount of modifiers, and easy to realize the roll-to-roll continuous production [255]. However, the bottleneck lies in the large-scale preparation of MXene, dispersion stability, and the high cost of two-dimensional materials. Future industrialization will require balancing process complexity, material cost, functional diversity, and environmental compatibility, while focusing on the development of key technologies such as needleless electrospinning, multi-nozzle arrays, and continuous post-processing to achieve both fine structural control and high production efficiency. In summary, the advantages of electrospinning technology lie in its ability to fabricate nanofibrous membranes with high specific surface area and high porosity, its strong process flexibility allowing precise control over fiber morphology and membrane structure by adjusting spinning parameters, and its ease of achieving multifunctional composites [256]. Compared with LBL self-assembly, electrospinning offers significant advantages in constructing three-dimensional network structures and achieving high-flux separation, and it is more amenable to scale-up. However, its precision in controlling the thickness and composition of functional layers is inferior to that of LBL self-assembly. This method is most suitable for preparing oil–water separation membranes requiring high flux and high separation efficiency, such as photo-responsive membranes [257], pH-responsive membranes, and fibrous membranes with photocatalytic self-cleaning functions, as well as nanofiber-based smart separation materials characterized by high porosity and rapid response [258]. Nevertheless, its limitations include the typically low mechanical strength of the fibrous membranes, and controlling the uniformity of fiber diameter during large-scale production remains a challenge.

### Surface-Initiated Atom-Transfer Radical Polymerization

The synergistic application of self-assembly technology and electrostatic spinning provides a new idea of multi-scale regulation for smart materials development, while more precise molecular-level design is required to further enhance the functionality and interfacial properties of materials. In this regard, atom transfer radical polymerization (ATRP) technology shows unique value as a highly controllable polymerization method that can precisely regulate the molecular structure and molecular weight distribution of polymers [259]. It ensures controlled chain growth during the polymerization process through reversible radical generation and trapping reactions [260], thus avoiding the broad molecular weight distribution common in traditional free radical polymerization.

**Fig. 13:**
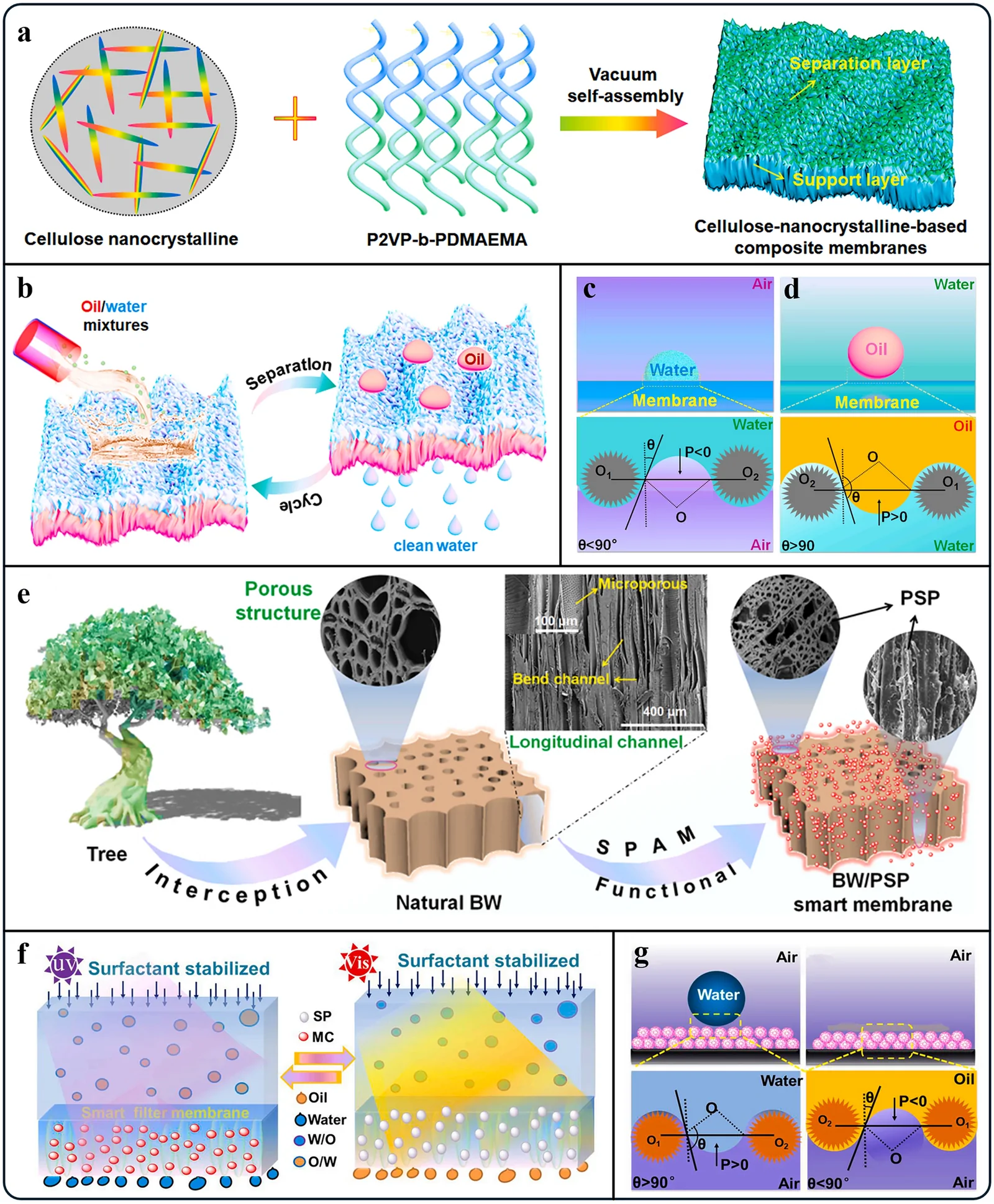
**a** Schematic diagram of the cellulose nanocrystalline-based composite membranes (CCM) preparation process. **b** Schematic diagram of oil–water mixture separation. The emulsion breaking process of CCM was analyzed. Wetting model of CCM, where **c** is a superhydrophilic osmosis model and **d** is an underwater superoleophobic osmosis model. Reproduced from Ref. [261] with permission, copyright 2023, Elsevier BV. **e** Preparation process of 3D smart BW/PSP membrane. Analysis of emulsion breaking mechanism of BW/PSP smart membrane. **f** Schematic diagram of separation process. **g** Wetting model of BW/PSP membrane. Reproduced from Ref. [262] with permission, copyright 2022, Elsevier BV

**Fig. 14:**
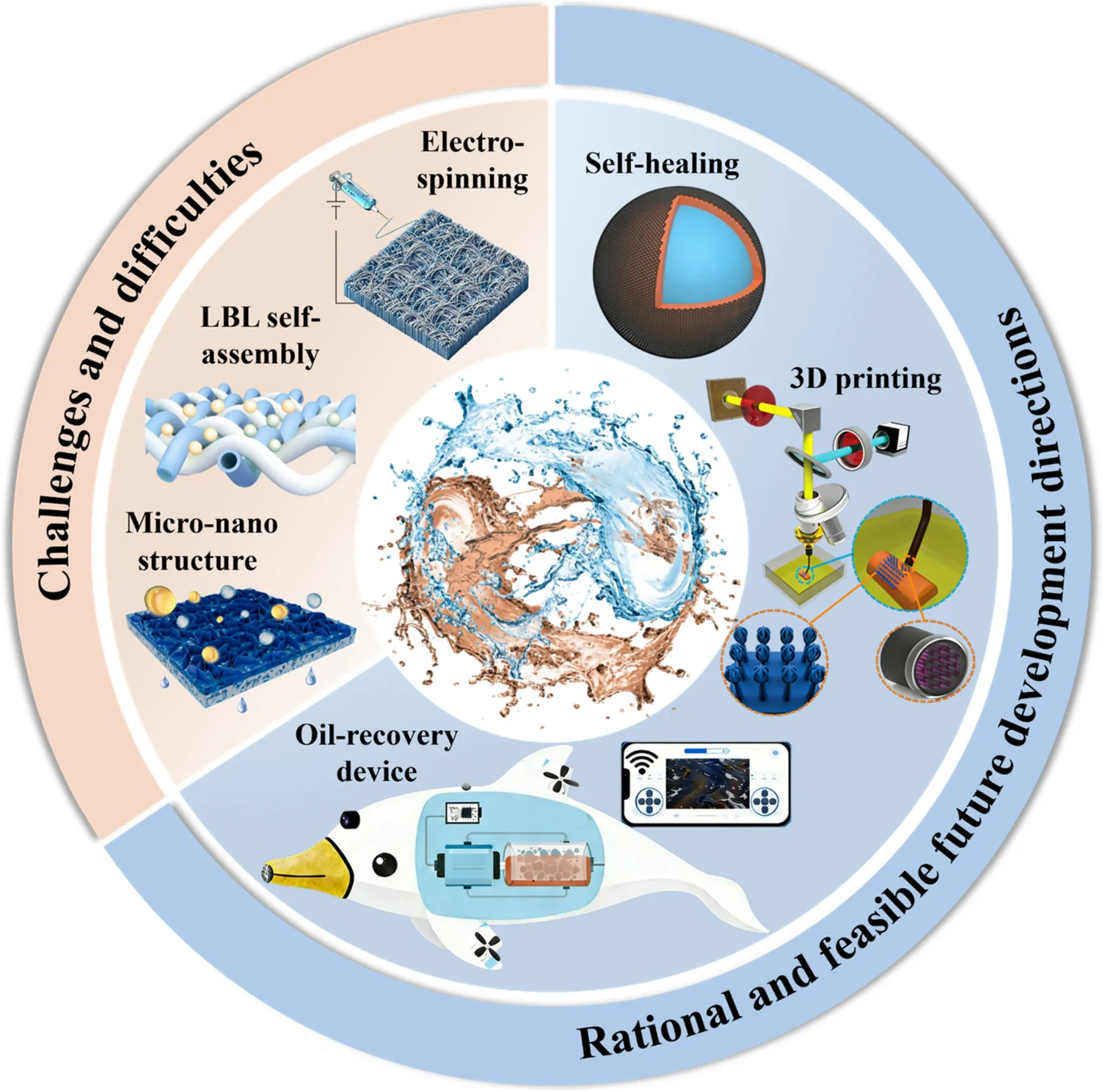
Current challenges and future development directions of smart-responsive wetting materials. Among these, the micro-/nanostructures are susceptible to damage in complex environments, leading to reduced durability. Reproduced from Ref. [280] with permission, copyright 2024, Elsevier BV. Secondly, existing preparation methods are often costly, pose significant environmental burdens, and are difficult to scale up. Reproduced from Ref. [281] with permission, copyright 2026, Elsevier BV. Reproduced from Ref. [256] with permission, copyright 2025, Elsevier BV. Future development should focus on three main directions: constructing self-healing micro-/nanostructures and exploring fluorine-free, low-surface-energy modifications; reproduced from ref. [282] with permission, copyright 2022, Spring Nature. Utilizing continuous production technologies like 3D printing and renewable resources such as biomass waste to develop bio-based materials; reproduced from Ref. [283] with permission, copyright 2017, Wiley. And deep integration with artificial intelligence, which is expected to drive breakthrough advancements in smart-responsive wetting materials. Reproduced from Ref. [284] with permission, copyright 2026, Wiley

ATRP has been widely used to synthesize polymers with a variety of topologies, such as block polymers, grafted polymers, star polymers, and hyperbranched polymers. In order to extend the applications of polymers, it is particularly important to combine functional polymers with substrate surfaces to construct smart-responsive surfaces. SI-ATRP provides a suitable method for precise surface modification by uniformly grafting functional polymer chains on the substrate surface, ensuring uniform and high-quality surface coverage while maintaining low polydispersity [263, 264]. As a result, SI-ATRP has become an important tool in the design of smart surfaces and functional materials, showing a wide range of applications. Cai et al. successfully prepared cellulose nanocrystal (CNC)-based composite membranes (CCMs) by SI-ATRP and vacuum self-assembly techniques (Fig. 13a). Cellulose nanocrystals have excellent biodegradability and high strength, but their pristine surface hydrophobicity limits the application. Its surface was modified by the SI-ATRP method to introduce functional polymer chains, which significantly improved the hydrophilicity of the membrane, while the cellulose nanocrystals were ordered by vacuum self-assembly technique to enhance the mechanical properties and surface characteristics of the membrane. The modified composite membrane exhibits excellent superhydrophilicity and superoleophobicity, with extremely low contact angle underwater and high water absorption ability; meanwhile, due to the special micro-/nanostructure on the surface, it is difficult for oil molecules to adhere to the membrane, which demonstrates excellent superoleophobicity (Fig. 13b). The membrane maintains its stability during long-term recycling and has good anti-pollution performance, which can effectively extend its service life. The CCM exhibits excellent performance in treating surfactant-stabilized O/W emulsions: it has a separation efficiency of more than 99.9%, a flux of 16,692 L m^−2^ h^−1^ bar^−1^, as well as good cyclic stability and anti-pollution properties (Fig. 13c, d) [261]. Such biomass-derived membranes are green, inexpensive, easy to fabricate, scalable, superwettable, and durable and are expected to be an alternative to separation membranes in today’s market. However, its surface wettability is relatively fixed, and its adaptability is limited when dealing with complex and changing or alternating types of oil and water systems. In contrast, Li et al. prepared three-dimensional (3D) smart membranes with switchable superhydrophobic/hydrophilic properties by grafting photo-responsive polyspiropyran (PSP) onto the surface of a wood substrate via surface ATRP (Fig. 13e). The membrane was able to realize hydrophilic–hydrophobic transition by switching between UV and visible light and possessed the ability to efficiently separate W/O and O/W emulsions, which was particularly suitable for oil–water separation. Compared with free spiropyran (SP), grafted PSP exhibits faster photo-response effect. Under UV and visible light irradiation, the membrane was able to rapidly modulate the surface properties to enhance the emulsion separation efficiency, especially in dynamic environments (Fig. 13f, g) [262]. Performance tests showed that the membrane has excellent separation capability, with a high flux of 4392 L m^−2^ h^−1^ for W/O emulsions and a separation efficiency of more than 99.99%. In addition, the membrane has good cyclic stability and maintains high separation efficiency after 12 cycles during 60 days of use with minimal performance degradation, providing a new idea for the design of functional biomass membranes (Fig. 14).

Although SI-ATRP can accurately construct functional polymer brushes, the scalability of its practical production is still a key bottleneck for industrial applications. Recent studies have demonstrated the potential of different pathways. Wang et al. grafted temperature-sensitive PNIPAM on melamine sponge as a substrate [265], with the advantages of low substrate cost and high oil absorption capacity of the three-dimensional structure, but with limited mechanical strength, and the reaction needs to be strictly anaerobic and time-consuming for hours, and the scale-up faces the challenges of homogeneity and efficiency. Yan et al. constructed micronanostructures by grafting polyacrylamides on PVDF membranes, with the highlights of ATRP controllability significantly improving hydrophilic and anti-fouling performance [266], but the process involves GO modification, silylation, initiator immobilization, and other multi-step reactions, the process is complex, catalyst residue, and solvent recovery to increase the cost of scale-up. Therefore, practical industrial application requires a trade-off between process complexity, reaction efficiency, cost, and functionality to achieve a balance between structural controllability and production efficiency [267, 268]. In summary, the advantage of the SI-ATRP technique lies in its ability to precisely graft polymer brushes with well-defined structures and narrow molecular weight distributions onto substrate surfaces, enabling molecular-level precision control over surface chemical properties. Compared with LBL self-assembly and electrospinning, SI-ATRP offers significant advantages in structural controllability at the molecular level, allowing for the attainment of the most uniform and well-defined surface chemistries. However, it is inferior to electrospinning in constructing three-dimensional porous structures [269]. This method is particularly suitable for preparing smart materials that require fine-tuning of surface functional groups and responsive behaviors, such as temperature-responsive, pH-responsive, and photo-responsive separation membranes, as well as responsive polymer brushes [270, 271]. Its limitations include relatively harsh reaction conditions, a time-consuming polymerization process, and technical bottlenecks in achieving uniform modification over large-area substrates and in scaling up for mass production. To provide a clearer and more intuitive comparison of the characteristics of the different preparation methods mentioned above, we have summarized their key features in a table. Detailed information can be found in Table 3.

**Table 3:** Comparison of three preparation methods

	LBL self-assembly	Electro-spinning	SI-ATRP
Process complexity	Requires alternating deposition, resulting in a relatively long fabrication cycle	Single-step film formation has higher efficiency but is more sensitive to the solution and process parameters	The reaction conditions are stringent, and the process chain is relatively long
Controllability	Precise control of film thickness, surface roughness, and chemical composition	Controllable fiber diameter, porosity, and surface roughness	Precise control of polymer brush chain length, grafting density, and surface functional groups
Cost	Low equipment requirements, but the time cost of multilayer assembly is relatively significant	The equipment is relatively mature, and the experimental cost is controllable	Higher requirements for initiators, catalysts, and process control, resulting in higher costs
Applicable substrates	Flat membranes, metal meshes, fibrous membranes, curved substrates, and porous substrates are all applicable	Suitable for polymers and their composite systems, and can also be deposited on supports such as metal meshes, nonwoven fabrics, and textiles	Applicable to substrates that can be surface-functionalized, such as PVDF membranes, wood, cellulose nanocrystals, and metal or oxide surfaces
Material structural characteristics	Multilayer composite films/functional coatings, enabling facile construction of surface functional gradients and multifunctional integration	Three-dimensional nanofiber network structures with high porosity and large specific surface area, which facilitate the formation of micro-/nanodual-scale rough surfaces	Surface-grafted polymer brush layers/nanoscale functional interfaces with uniform coverage and well-defined structures
Refs	[63, 239–242, 244]	[246, 250, 256, 258]	[259, 261, 262, 270, 271]

### Other Methods

In addition, a variety of other methods have been widely used for the preparation of smart-responsive surfaces, such as sol–gel method [272], photolithography [273], femtosecond laser [274], chemical vapor deposition [275], atomic layer deposition [276], nonsolvent-induced phase separation coupled with in situ mineralization [226], and electrochemical deposition [277]. Among them, the sol–gel electrochemical deposition technique has a unique advantage: this method can locally and selectively accelerate the conventional sol–gel process by constructing a special high pH chemical microenvironment at the electrode interface, thus realizing the rapid, uniform, dense, and strongly bonded deposition of functional materials on the electrode surface. Wang et al. successfully prepared UV-responsive TiO_2_ on cellulose substrate based on this strategy. TiO_2_/silane composite films on cellulose substrate with UV-responsive properties are realizing reversible switching between superhydrophobic and superhydrophilic states [278]. Under the optimal process conditions (addition of 2% octyltriethoxysilane, deposition at 13 V for 8 min), the contact angle of the resulting coatings was as high as 166.1°, which exhibited excellent superhydrophobicity. After irradiation with high-intensity UV light (10^5^ μW cm^−2^) for 11 min, the surface rapidly transformed into a superhydrophilic state (contact angle of 0°), and the contact angle could be restored to 150.6° after 40 h of resting in a dark environment, which showed good reversibility. In addition, the coating significantly enhances the bonding strength with the substrate through electrochemical deposition and shows excellent washing resistance, with the contact angle remaining at 119.1° after 10 washes, providing a new technical path for the development of high-performance smart textiles and functional coatings. In contrast, femtosecond laser processing represents another efficient physical fabrication strategy, offering a green and efficient new pathway for the preparation of smart wetting materials. Li et al. combined a metal mesh with cured PDMS and utilized one-step femtosecond laser ablation to construct multi-level micro-groove structures on the metal wire surface [279]. Concurrently, the laser induced the vaporization and decomposition of PDMS into hydrophobic SiO_2_ nanoparticles, which were deposited in situ within the grooves, achieving the one-step completion of roughness construction and low-surface-energy modification. After O_2_ plasma treatment, this surface reversibly transitioned to a superhydrophilic/underwater superoleophobic state. Heating at 80 °C could restore its superhydrophobicity, enabling on-demand oil–water separation with an efficiency exceeding 99.2%. This method is entirely fluorine-free and free of chemical reagents, and the resulting surface can withstand abrasion by sandpaper over a distance of 100 m under a 500-g load, as well as high-temperature treatment up to 400 °C. This provides a new strategy for the green preparation of highly durable smart separation membranes.

## Conclusion and Outlook

The evolution of oil–water separation technology clearly reveals a profound transition in separation mechanisms, from macroscopic force fields to microscopic interfaces, alongside an expansion of separation functions from single to multiple modes. This progression began with passive physical separation reliant on density differences (e.g., gravity settling), advanced to dynamically enhanced separation through the introduction of external energy (e.g., centrifugation, coalescence), and then, leveraging bionic principles, achieved a leap from “force-field driven” to “interfacial chemical regulation” through superwetting materials, thereby accomplishing the diversification of separation functions. Today, smart wettability materials have triggered a profound paradigm shift in this field. They have reshaped separation materials from passive tools with fixed properties into active, intelligent platforms. These platforms are capable of sensing environmental stimuli (e.g., pH and temperature) and dynamically switching their wettability, thereby enabling adaptive separation and self-cleaning. This paradigm shift is the central focus of this review. The research progress of smart-responsive wettability materials is reviewed in this paper. First, based on previous studies, the theoretical basis of wettability and the mechanism of oil–water separation are discussed. Subsequently, the structural characteristics and separation performance of four types of special wettability materials are comparatively analyzed. Focusing on eight categories of smart-responsive materials (temperature, pH, light, electricity, gas, ion, solvent, and multi-response), the response mechanisms, application advantages, and limitations of each material type in oil–water separation are analyzed in depth. In addition, key preparation techniques, including LBL self-assembly, electrospinning, and surface-initiated atom transfer radical polymerization, are compared in terms of their advantages and disadvantages. Finally, the current status and remaining challenges in the field of smart-responsive wettability materials are summarized, and future development directions are discussed.

Despite significant progress in laboratory research, smart-responsive materials still face three core challenges in their transition toward practical applications. First, in terms of durability, the micro- and nanostructures are prone to damage in complex environments, and the reversibility of the responsive behavior tends to degrade over time [280]. Second, regarding the preparation process, existing methods are often costly, environmentally burdensome, and difficult to scale up [256, 281]. Third, in terms of response mechanisms, single-stimulus responsiveness is insufficient to cope with the dynamic complexity of industrial wastewater. Recent studies have provided important explorations to bridge the gap between laboratory research and industrial applications from three key dimensions [285–287]. Wang et al. prepared a large-sized CO_2_-responsive membrane with an area of 3,600 cm^2^ [185], advancing the membrane scale toward the pilot level. Luo et al. verified the environmental durability of diatomaceous earth coatings through salt spray, artificial seawater, and UV aging tests under simulated marine conditions [288]. Feng et al. designed a multifunctional membrane that achieved 99.9% separation efficiency and 97.6% dye removal in real dyeing wastewater [289]. However, field pilot tests and long-term operational validation remain the final obstacles before industrialization.

Looking to the future, the development of smart response wetting materials should focus on three major directions:

Constructing self-healing micro- and nanostructures, exploring fluorine-free low-surface-energy modification [282], and introducing multi-responsive groups to achieve synergistic modulation. This will help develop low-cost and highly durable multi-responsive materials.

Developing solvent-free/aqueous phase synthesis processes, drawing on continuous production technologies such as roll-to-roll coating and 3D printing [283, 290, 291], and using renewable resources such as biomass waste to develop bio-based materials. This is conducive to promoting green manufacturing and large-scale production.

Deep integration of artificial intelligence is expected to promote the breakthrough development of smart response wettability materials [292]. First, by establishing a wettability database and experimental prediction model, the target molecular structure and micro-/nano-configuration can be quickly screened [293]. Second, a closed-loop system of intelligent sensing and adaptive regulation is constructed [284], integrating microsensors to monitor key parameters such as water quality, oil droplet size, and membrane contamination in real time and dynamically optimizing the separation mode with the help of AI algorithms [294]. Finally, process simulation technology can build cross-scale models to simulate the transport and separation behavior of complex oil–water systems [295], providing a theoretical basis for industrial device design.

In summary, smart response wetting materials are experiencing a profound paradigm shift from static to dynamic, from single to multiple, and from passive to intelligent. Focusing on the three major directions of material performance optimization, preparation process innovation, and AI technology empowerment, and continuing to promote the synergistic innovation between basic research and application, smart response wetting materials are expected to truly move from the laboratory to practical application and to contribute to the protection of global water resources.
